# Dynamic Regulation of Genes Involved in Mitochondrial DNA Replication and Transcription during Mouse Brown Fat Cell Differentiation and Recruitment

**DOI:** 10.1371/journal.pone.0008458

**Published:** 2009-12-24

**Authors:** Maria Murholm, Karen Dixen, Klaus Qvortrup, Lillian H. L. Hansen, Ez-Zoubir Amri, Lise Madsen, Giorgio Barbatelli, Bjørn Quistorff, Jacob B. Hansen

**Affiliations:** 1 Department of Biomedical Sciences, The Panum Institute, University of Copenhagen, Copenhagen, Denmark; 2 IBDC, Université de Nice Sophia-Antipolis, CNRS, UMR 6543, Nice, France; 3 Department of Biology, University of Copenhagen, Copenhagen, Denmark; 4 National Institute of Nutrition and Seafood Research, Bergen, Norway; 5 Department of Molecular Pathology and Innovative Therapies, School of Medicine, University of Ancona, Ancona, Italy; Fundação Oswaldo Cruz, Brazil

## Abstract

**Background:**

Brown adipocytes are specialised in dissipating energy through adaptive thermogenesis, whereas white adipocytes are specialised in energy storage. These essentially opposite functions are possible for two reasons relating to mitochondria, namely expression of uncoupling protein 1 (UCP1) and a remarkably higher mitochondrial abundance in brown adipocytes.

**Methodology/Principal Findings:**

Here we report a comprehensive characterisation of gene expression linked to mitochondrial DNA replication, transcription and function during white and brown fat cell differentiation *in vitro* as well as in white and brown fat, brown adipose tissue fractions and in selected adipose tissues during cold exposure. We find a massive induction of the majority of such genes during brown adipocyte differentiation and recruitment, e.g. of the mitochondrial transcription factors A (Tfam) and B2 (Tfb2m), whereas only a subset of the same genes were induced during white adipose conversion. In addition, PR domain containing 16 (PRDM16) was found to be expressed at substantially higher levels in brown compared to white pre-adipocytes and adipocytes. We demonstrate that forced expression of Tfam but not Tfb2m in brown adipocyte precursor cells promotes mitochondrial DNA replication, and that silencing of PRDM16 expression during brown fat cell differentiation blunts mitochondrial biogenesis and expression of brown fat cell markers.

**Conclusions/Significance:**

Using both *in vitro* and *in vivo* model systems of white and brown fat cell differentiation, we report a detailed characterisation of gene expression linked to mitochondrial biogenesis and function. We find significant differences in differentiating white and brown adipocytes, which might explain the notable increase in mitochondrial content observed during brown adipose conversion. In addition, our data support a key role of PRDM16 in triggering brown adipocyte differentiation, including mitochondrial biogenesis and expression of UCP1.

## Introduction

White and brown fat cells share a number of characteristics, including similarities in cell morphology, secretion of adipokines, enzymes of lipid metabolism and patterns of gene expression [1], [2]. Despite these similarities, white and brown adipose tissues (WAT and BAT, respectively) carry out essentially opposite functions, with WAT being the major energy reserve through triglyceride accumulation, and BAT having the ability to dissipate energy through adaptive thermogenesis. The powerful energy dissipating and thermogenic capacity of BAT is due to two mitochondrial features of brown adipocytes: the presence of uncoupling protein 1 (UCP1) and a high number of mitochondria, which considerably exceeds that of white fat cells [1]–[3]. UCP1 is expressed exclusively in brown fat cells. Recently, it was demonstrated that white and brown fat cells do not develop from a common precursor, as BAT depots but not WAT seems to arise from precursor cells shared with skeletal muscle [4], [5]. Intriguingly, brown-like adipocytes appearing in some WAT depots following extended periods of exposure to a β-adrenergic agonist do not share progenitors with skeletal muscle [4]. These observations suggest that two types of brown fat cells could exist in mammals.

The mitochondrial genome (mtDNA) is a circular, double-stranded molecule of 16,300 bp in mice. It is present in multiple copies in each mitochondrion and a mammalian cell contains between 1,000 and 10,000 copies [6]–[8]. mtDNA encodes key components of the electron transport chain as well as RNA components required for mitochondrial translation. The mitochondrial replication machinery is encoded by nuclear genes, the products of which translocate to the mitochondrion. These include the mtDNA polymerase γ (Polg), single strand-binding protein (Ssb), the replicative mitochondrial helicase (Twinkle) and the mitochondrial RNA processing RNase (RNase MRP) [7].

Mitochondrial transcription is controlled by nucleus-encoded transcription regulators that localize to either the nucleus or the mitochondrion. Key among the former are the nuclear respiratory factor 1 (NRF-1) and NRF-2 [called GA repeat-binding protein (GABP) in the mouse], which control the expression of a large number of genes important for mitochondrial respiration and translation as well as mtDNA replication and transcription [6], [8]. Nucleus-encoded transcription factors translocating to the mitochondrion include the mitochondrial RNA polymerase (PolRMT), mitochondrial transcription factors A, B1 and B2 (Tfam, Tfb1m, and Tfb2m, respectively) and the mitochondrial transcription termination factor 1 (mTERF1) [7], [9]. Three more mTERF genes have been identified (mTERF2-4) [10]–[12]. Finally, Tfam is not only crucial for mitochondrial transcription, but is also a positive regulator of mtDNA copy number [13].

The regulation of mitochondrial biogenesis during adipocyte differentiation in general and the molecular background for the difference in abundance of mitochondria in white and brown fat cells is not fully understood, but the observation that levels of peroxisome proliferator-activated receptor γ (PPARγ) co-activator 1α (PGC-1α) and PGC-1β are substantially higher in the latter strongly suggest their involvement. This has subsequently been confirmed in brown adipocytes *in vitro* and *in vivo*, where it was found that simultaneous loss of PGC-1α and PGC-1β attenuates mitochondrial biogenesis, whereas loss of only one has little effect [14], [15]. In addition, forced expression of PGC-1α in white adipocytes causes a transition towards a brown-like fat cell phenotype [16]–[18]. However, the mechanism by which PGC-1α and PGC-1β enforces mitochondrial biogenesis in brown fat cells is not fully explored.

Besides PGC-1α and PGC-1β, a number of nuclear transcription regulators have been reported to modulate mitochondrial biogenesis in adipocytes and/or adipose tissue, including PR domain containing 16 (PRDM16) [19], receptor interacting protein 140 (RIP140) [20], estrogen-related receptor α (ERRα) [21], small heterodimer partner (SHP) [22] and retinoblastoma protein (pRB) [23], [24]. The effects of most of these have been linked directly or indirectly to the function of PGC-1 family members, e.g. PRDM16 that has been demonstrated to promote mitochondrial biogenesis and BAT-selective gene expression in adipocytes, at least in part by increasing activities of PGC-1α and PGC-1β, and additionally via direct binding and activation of the PGC-1α promoter [19], [25].

Here we investigate the regulation of several aspects of mitochondrial function during adipocyte differentiation, with emphasis on brown adipogenesis *in vitro* and recruitment of brown fat cells in cold-challenged mice. We demonstrate that the majority of factors involved in mitochondrial transcription and replication are up-regulated during brown adipose conversion, whereas many of the same genes remain unchanged or are induced to a lesser extent during white adipocyte differentiation. In addition, we provide evidence that overexpression of Tfam or knockdown of PRDM16 influences mitochondrial DNA replication, gene expression and biogenesis.

## Materials and Methods

### Animals and Tissues

Interscapular BAT (iBAT) and epididymal WAT (eWAT) were obtained from a male C57BL/6J mouse. The stromal-vascular (SVF) and adipocyte (AF) fractions were obtained from twelve 10-week-old C57BL/6J mice (Charles River) that were maintained under constant temperature (21±2°C) and 12∶12-h light-dark cycles with ad libitum access to standard chow diet and water. Animals were killed by cervical dislocation, and interscapular fat depots were rapidly excised and washed in phosphate-buffered saline, minced and digested for 45 min at 37°C with 2 mg/ml collagenase A (Roche) in the presence of 20 mg/ml bovine serum albumin (Sigma) in Dulbecco's modified Eagle's medium (DMEM) under mild agitation. After addition of fetal bovine serum (FBS), adipocytes were separated from stromal-vascular cells by filtration (250, 80 and 25 µm nylon filters) and centrifugation as described previously [26], [27]. Contaminating erythrocytes were eliminated from the SVF by incubation in red blood cell lysis buffer (Sigma). Total RNA was extracted from each of the two pooled fractions as described below. For the cold-challenge experiment, female mice of the Sv129 strain were used as described [28], [29]. Mice acclimated at 28°C were subsequently maintained at 28°C or placed at 6°C for 1 or 3 days, at which time interscapular and inguinal adipose tissues were dissected. Experimental groups included 4 mice. Animal procedures and care were in accordance with Italian National Institute of Medical Research guidelines approved by The European Convention for the protection of vertebrate animals used for experimental and other scientific purposes.

### Cell Culture and Packaging of Virus

Wild-type and retinoblastoma gene-deficient (Rb−/−) mouse embryo fibroblasts (MEFs) were propagated and differentiated as previously described [30] with minor modifications. Briefly, MEFs were grown in AmnioMax C-100 Basal Medium (Invitrogen) supplemented with 7.5% FBS, 7.5% AmnioMax-C100 Supplement and 2 mM glutamine. The medium was changed every other day. For differentiation, 1-day postconfluent cells (designated day 0) were treated with growth medium containing 1 µM dexamethasone (Sigma), 0.5 mM methylisobutylxanthine (Sigma), 5 µg/ml insulin (Roche) and 0.5 µM rosiglitazone (kindly provided by Novo Nordisk) for 2 days. From day 2, the medium contained 5 µg/ml insulin and 0.5 µM rosiglitazone and was changed every other day. The 3T3-L1 white pre-adipocyte cell line [31] was grown to confluence in DMEM containing 10% bovine serum. Two-days postconfluent cells (day 0) were induced to differentiate with DMEM containing 10% FBS, 1 µM dexamethasone, 0.5 mM methylisobutylxanthine, 5 µg/ml insulin and 0.5 µM rosiglitazone. At days 2, 4 and 6, the cells were refed with DMEM containing 10% FBS supplemented with 5 µg/ml insulin and 0.5 µM rosiglitazone. The WT-1 pre-adipocyte cell line [32] was kindly provided by Dr. C. Ronald Kahn and was established by immortalisation of primary brown pre-adipocytes from newborn pups with simian virus 40 large T antigen. WT-1 cells were propagated and differentiated in DMEM supplemented with 10% FBS. For differentiation, 1-day postconfluent cells were treated with the same adipogenic compounds as described above for 3T3-L1 cells. Phoenix cells were cultured in DMEM with 10% FBS, and packaging and use of retrovirus were performed as previously described [33]. Transduced cells were selected with 8 µg/ml blasticidin S HCl (Invitrogen), 5 µg/ml puromycin (Sigma) or 200 µg/ml G418 (Sigma). All media described above contained 62.5 µg/ml penicillin and 100 µg/ml streptomycin, and all cells were cultured in a humidified atmosphere of 5% CO_2_ at 37°C.

### Plasmids

The retroviral vectors pMSCVneo and pMSCVpuro were from Clontech and pMSCVbsd was kindly provided by Dr. Reuven Agami [34]. To improve the multiple cloning site (MCS) of pMSCVbsd (and pMSCVpuro), the original *Hin*dIII site between the PGK promoter and the blasticidin (or puromycin) resistance gene was destroyed and two partially annealed oligos (5′-GATCTGTTTAAACGTCGACCCATGGGGATCCAAGCTTCCTGCAGGGCGGCCGCGGGCCCC and 5′-TCGAGGGGCCCGCGGCCGCCCTGCAGGAAGCTTGGATCCCCATGGGTCGACGTTTAAACA) were ligated into the *Bgl*II/*Xho*I site of the MCS, thereby creating pMSCVbsd link3 (or pMSCVpuro link3) having a new MCS with the following unique restriction enzyme sites: *Bgl*II-*Pme*I-*Sal*I-*Nco*I-*Bam*HI-*Hin*dIII-*Sbf*I-*Not*I-*Apa*I-*Xho*I-*Hpa*I-*Eco*RI. To improve the MCS of pMSCVneo, the original *Bam*HI, *Sal*I and *Hin*dIII sites between the neomycin resistance gene and the 3′ long terminal repeat were destroyed and the two partially annealed oligos described above were ligated into the *Bgl*II/*Xho*I site of the MCS, thereby creating pMSCVneo link3 having a new MCS with the following unique restriction enzyme sites: *Eco*RI-*Hpa*I-*Xho*I-*Apa*I-*Not*I-*Sbf*I-*Hin*dIII-*Bam*HI-*Sal*I-*Pme*I-*Bgl*II. The pm-TFAM-HA vector encoding a HA-tagged full-length mouse Tfam was obtained from Dr. Dongchon Kang [35]. The insert was excised with *Bam*HI/*Not*I and ligated into the *Bam*HI/*Not*I site of pMSCVpuro link3 and pMSCVbsd link3, thereby creating the vectors pMSCVpuro-Tfam-HA and pMSCVbsd-Tfam-HA. The Tfb2m-His-pBacPAK8 vector encoding full-length His-tagged mouse Tfb2m was kindly provided by Dr. Claes M. Gustafsson [36]. The Tfb2m insert was excised with *Xho*I/*Not*I and ligated into the *Xho*I/*Not*I site of pMSCVneo link3, thereby creating pMSCVneo-Tfb2m-His. pSUPER.retro.neo was obtained from OligoEngine and pSUPER.retro.neo-PRDM16 has been described [19] and was purchased from Addgene (Addgene plasmid 15505).

### RT-qPCR

Total RNA was purified from cells and tissues using TRIzol (Invitrogen) or Tri-Reagent (Euromedex). Reverse transcriptions were performed in 25 µl reactions containing 1x 1st Strand Buffer (Invitrogen), 2 µg random hexamers (GE Healthcare), 0.9 mM of each dNTP (GE Healthcare), 20 units of RNAguard (GE Healthcare), 1 µg of total DNase-treated RNA and 200 units of Moloney murine leukaemia virus reverse transcriptase (Invitrogen). Reactions were left for 10 min at room temperature, followed by incubation at 37°C for 1 h. After cDNA synthesis, reactions were diluted with 50 µl of water and frozen at −80°C. cDNA was analysed by reverse transcription-quantitative polymerase chain reaction (RT-qPCR) using the Stratagene Mx3000P QPCR System. Each PCR mixture contained, in a final volume of 20 µl, 1.5 µl of first-strand cDNA, 10 µl of Brilliant QRT-PCR Master Mix (Stratagene) and 2 pmol of each primer. PCR primers are listed in Table S1. All reactions were performed using the following cycling conditions: 95°C for 10 min, then 40 cycles of 95°C for 15 s, 55°C for 30 s and 72°C for 15 s. PCR was carried out in 96-well plates and each sample was run in duplicate. Target gene mRNA expression was normalized to expression of TATA-binding protein (TBP) mRNA or 18S ribosomal RNA (rRNA). Absolute values of samples normalised to TBP (gene of interest/TBP) are provided in Table S2 to allow comparison of expression levels across cell lines and tissues.

### Quantification of Relative mtDNA Copy Numbers

Cells were washed in phosphate-buffered saline, scraped off the plates in lysis buffer containing 100 mM Tris-base (pH 8.0), 5 mM EDTA (pH 8.0), 0.2% sodium dodecyl sulphate, 200 mM NaCl and 100 µg/ml proteinase K and incubated overnight at 55°C with rotation. DNA was precipitated with two volumes of 99% ethanol and fished out with inoculation loops, washed in 70% ethanol and dissolved in 1xTE buffer containing 10 µg/ml RNase A at 55°C overnight. Two dishes were harvested at each time point. DNA concentrations were determined on the Eppendorf BioPhotometer at 260 nm and 50 ng DNA was used for qPCR. Primers sets used were against COX II (mtDNA) and RIP140 (nuclear DNA) (Table S1).

### Citrate Synthase Activity

Cells were harvested in GG-buffer (pH 7.5) containing 25 mM glycyl-glycin, 150 mM KCl, 5 mM MgSO_4_ and 5 mM EDTA as well as freshly added DTT (1 mM), BSA (0.02%) and Triton X-100 (0.1%), vortexed and frozen in liquid nitrogen. Two dishes were harvested at each time point. Samples were thawed on ice and centrifuged at 4°C at 20.000 g for 2 minutes. Supernatants were used for measurements. Citrate synthase activity was measured spectrophotometrically at 25°C and 412 nm. Citrate synthase buffer contained 100 mM Tris-base (pH 8.0), 10 mM DTNB, 5 mM acetyl-CoA and 50 mM oxaloacetic acid [37]. Each sample was measured in duplicate and the mean was used for subsequent calculations. Activities were normalized to the amount of protein determined by the Lowry method [38].

### Transmission Electron Microscopy

Cells were washed in 37°C 0.15 M Sorensens Phosphate Buffer (pH 7.4) (Electron Microscopy Sciences) and subsequently fixed in 2% glutaraldehyde in 0.05 M Sorensens Phosphate Buffer while rotating overnight at 4°C. The samples were rinsed three times in 0.15 M Sorensens Phosphate Buffer (pH 7.4) and subsequently postfixed in 1% OsO_4_ in 0.12 M sodium cacodylate buffer (pH 7.4) for 2 h. The specimens were dehydrated in graded series of ethanol, transferred to propylene oxide and embedded in Epon according to standard procedures. Ultra thin sections were cut with a Reichert-Jung Ultracut E microtome and collected on single slot copper grids with Formvar supporting membranes. The sections were stained with uranyl acetate and lead citrate and examined with a Philips CM 100 transmission electron microscope, operated at an accelerating voltage of 80 kV and equipped with a SIS MegaView2 camera. Digital images were recorded with the analySIS software package.

### Statistical Analyses

Time-course studies with wild-type and Rb−/− MEFs were analysed for statistical significance (p<0.05) using multiple linear regression of means using PROC REG (SAS 9.1.2, SAS Institute) with expression level as the dependent variable and cell type and time as independent variables. It was assumed that residual variance was identical for the two cell types. A difference between means was considered statistically significant if there was no overlap between their 95% confidence intervals. All other relevant data were analysed for statistical significance (p<0.05) using Student's t-test (Microsoft Office Excel). Statistical analysis of data for eWAT and iBAT was not possible, as the tissues were from a single mouse. Similarly, statistics were not conducted on BAT fractions, as the measurements were performed on pools of RNA from 12 mice.

## Results

### Expression of Brown Adipose-Selective Genes during Differentiation of White and Brown Adipocytes

We aimed at characterising various aspects of mitochondrial biogenesis and function during fat cell differentiation, with emphasis on differences between white and brown adipocytes. Therefore, we initially measured expression levels of genes involved in mitochondrial DNA replication, transcription and function during adipogenesis of mouse fibroblasts and pre-adipocytes capable of differentiation to white or brown fat cells. We reported previously that wild-type mouse embryo fibroblasts (MEFs) differentiate to white fat cells, whereas MEFs lacking a functional retinoblastoma gene (designated Rb−/− MEFs) differentiate to brown adipocytes [23]. Wild-type and Rb−/− MEFs were subjected to a time-course study. In addition, we used two pre-adipocyte cell lines, namely 3T3-L1 white and WT-1 brown pre-adipocytes as supplemental model systems to validate expression patterns of selected genes. Gene expression was determined by quantitative RT-PCR (RT-qPCR) and presented as indicated in the figure legends. Table S2 provides the normalised expression of all genes measured to allow comparison of expression levels across cell lines and tissues.

**Figure 1:**
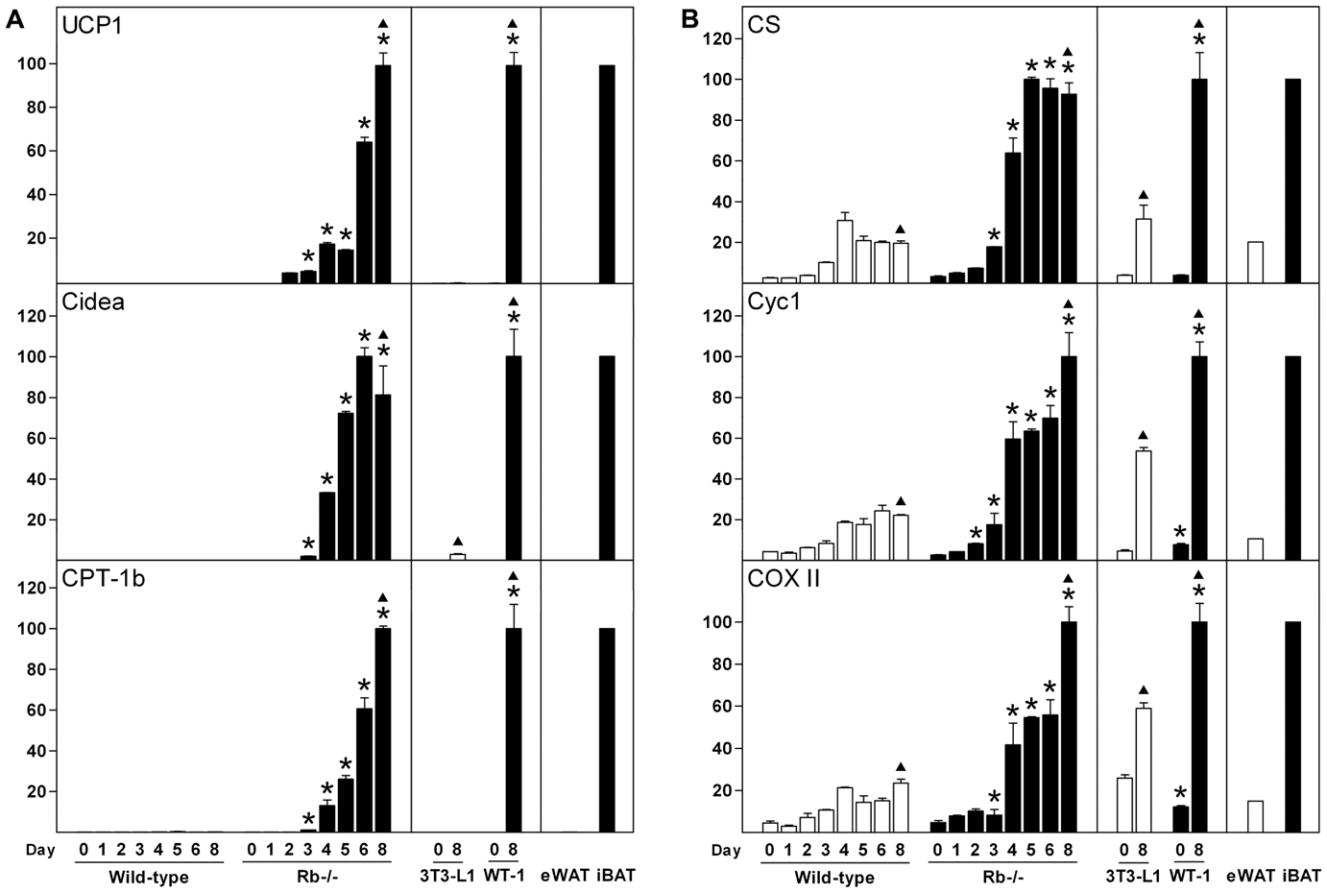
Expression of brown fat-selective genes and genes indicative of differential mitochondrial biogenesis during differentiation of white and brown adipocytes. Cell lines were induced to differentiate as described in “Materials and Methods” and total RNA was harvested at the indicated days of differentiation. In addition, RNA from eWAT and iBAT was included. Expression levels were determined by RT-qPCR and relative expression levels of genes indicated in the figure determined by normalisation to the levels of TBP. In each of the three boxes for the individual genes, the mean of the normalized expression level of the sample with the highest value was set to 100. Error bars represent SEM. (**A**) Expression of the brown adipose-selective genes UCP1, Cidea and CPT-1b. (**B**) Gene expression pattern indicative of differential mitochondrial biogenesis. Genes measured were CS, Cyc1 and COX II. Results from one of two independent cell culture experiments are shown. *, p<0.05 [day X in wild-type MEFs (or 3T3-L1) compared to day X in Rb−/− MEFs (or WT-1)]. ▴, p<0.05 (day 0 vs. day 8 for each of the four cell lines).

Wild-type MEFs, Rb−/− MEFs, 3T3-L1 and WT-1 cells efficiently differentiated to adipocytes following adipogenic stimulation at day 0, as demonstrated by robust induction of adipocyte marker genes like PPARγ2, CCAAT/enhancer-binding protein α (C/EBPα), adiponectin and fatty acid-binding protein 4 (FABP4, also called aP2) (as determined by RT-qPCR) as well as accumulation of lipid droplets in >90% of the cells (Fig. S1 and data not shown). Cells were considered as mature adipocytes at day 8. The general marker genes were expressed at equal levels in epididymal WAT (eWAT) and interscapular BAT (iBAT), except for adiponectin, which was enriched in the former (Fig. S1). eWAT was chosen as WAT depot in this case, as it contains very few brown adipocytes and is refractory to cold-induced recruitment of brown-like adipocytes [39]. Conversely, brown adipose-specific or -enriched genes like UCP1, cell death-inducing DFF45-like effector A (Cidea) [40] and carnitine palmitoyltransferase 1b (CPT-1b) were selectively induced in differentiating Rb−/− MEFs and WT-1 cells compared to differentiating wild-type MEFs and 3T3-L1 pre-adipocytes (Fig. 1A). Levels of UCP1 mRNA in Rb−/− and WT-1 adipocytes were 5–10 times lower than in iBAT, but 3000–6000 times higher than in eWAT (Table S2). Most markers, whether general adipose markers or brown fat-enriched markers, started to increase around day 2 or 3. To probe whether the cell lines becoming brown adipocytes, like primary brown pre-adipocytes [4], [5], expressed markers of skeletal muscle before differentiation, we measured the level of selected skeletal muscle markers at day 0. Consistent with their brown adipogenic fate, myogenin mRNA was present at higher levels in Rb−/− and WT-1 cells compared to wild-type MEFs and 3T3-L1 cells (Fig. S2). Of interest, myogenin was expressed at substantially higher levels in WT-1 pre-adipocytes compared to Rb−/− MEFs (Table S2), possibly reflecting their different origins as primary brown pre-adipocytes and embryo fibroblasts, respectively. Of notice, expression of MyoD and Myf-5 was not enriched in Rb−/− MEFs and WT-1 pre-adipocytes (data not shown).

### Changes in Gene Expression, Enzyme Activities and mtDNA Copy Number Indicative of Differential Mitochondrial Biogenesis during Differentiation of White and Brown Fat Cells *In Vitro*

**Figure 2:**
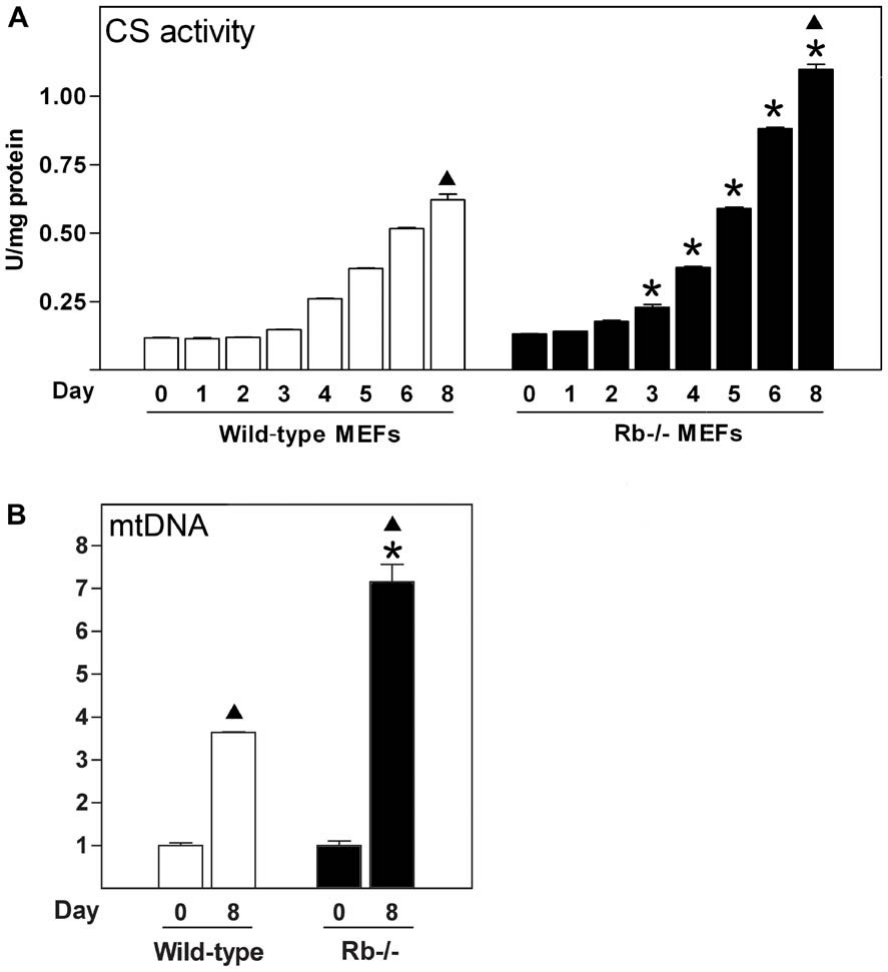
Citrate synthase activity and relative mtDNA copy number during white and brown fat cell differentiation. Cell lines were induced to differentiate as described in “Materials and Methods” and cells were harvested for measurements of enzymatic activities or relative mtDNA copy numbers at the indicated days of differentiation. Error bars represent SEM. (**A**) Enzyme activity of CS was determined as described in “Materials and Methods”. Enzyme activities (U) were normalised to the concentration of protein. (**B**) Relative mtDNA copy number was determined by qPCR using primer sets specific for mtDNA (COX II) and nDNA (RIP140), and relative mtDNA levels were calculated by normalising signals from COX II to those of RIP140. The mean of the normalized values for wild-type cells on day 0 was set to 1. A representative of three independent experiments is shown. *, p<0.05 (day X in wild-type MEFs compared to day X in Rb−/− MEFs). ▴, p<0.05 (day 0 vs. day 8 for wild-type or Rb−/− MEFs).

Citrate synthase (CS) has been used in adipose tissue as a measure of mitochondrial respiratory chain activity and mitochondrial biogenesis [41], [42]. CS mRNA levels increase during differentiation of all four differentiation models used here, but are induced to 3–4-fold higher levels in Rb−/− and WT-1 cells compared to wild-type MEFs and 3T3-L1 cells (Fig. 1B). Similarly, CS enzyme activity was increased during adipose conversion of both wild-type and Rb−/− MEFs, with CS reaching a higher activity in the latter (Fig. 2A). Of notice, the difference in CS activity between wild-type and Rb−/− fat cells was smaller than the difference observed for CS mRNA levels. Expression of two electron transport chain components, cytochrome c-1 (Cyc1) and cytochrome c oxidase II (COX II), was induced to ∼4-fold higher levels in differentiating Rb−/− compared with wild-type MEFs (Fig. 1B). Cyc1 displayed a similar fold induction during differentiation of WT-1 and 3T3-L1 cells, although the absolute level was ∼2-fold higher in the former (Fig. 1B). COX II was induced ∼6-fold in WT-1 and ∼2-fold in 3T3-L1 cells. Consistently, CS, Cyc1 and COX II mRNAs were enriched in iBAT relative to eWAT. To probe whether the increased level and activity of CS were accompanied by changes in mtDNA copy number, we determined the ratio of mtDNA and nuclear DNA (nDNA) by qPCR before differentiation was initiated (day 0) and in the mature adipose state (day 8). The mtDNA/nDNA ratio increased 3.5-fold in wild-type cells and 7-fold in Rb−/− cells, suggesting that more mtDNA replication occurred during brown compared with white adipose conversion (Fig. 2B). In summary, the gene expression pattern in Rb−/− and WT-1 brown adipocytes indicates a more pronounced mitochondrial biogenesis than in wild-type MEF-derived and 3T3-L1 white adipocytes.

### Transmission Electron Microscopy Demonstrates Mitochondrial Biogenesis during Adipose Conversion of White and Brown Fat Cells *In Vitro*

**Figure 3:**
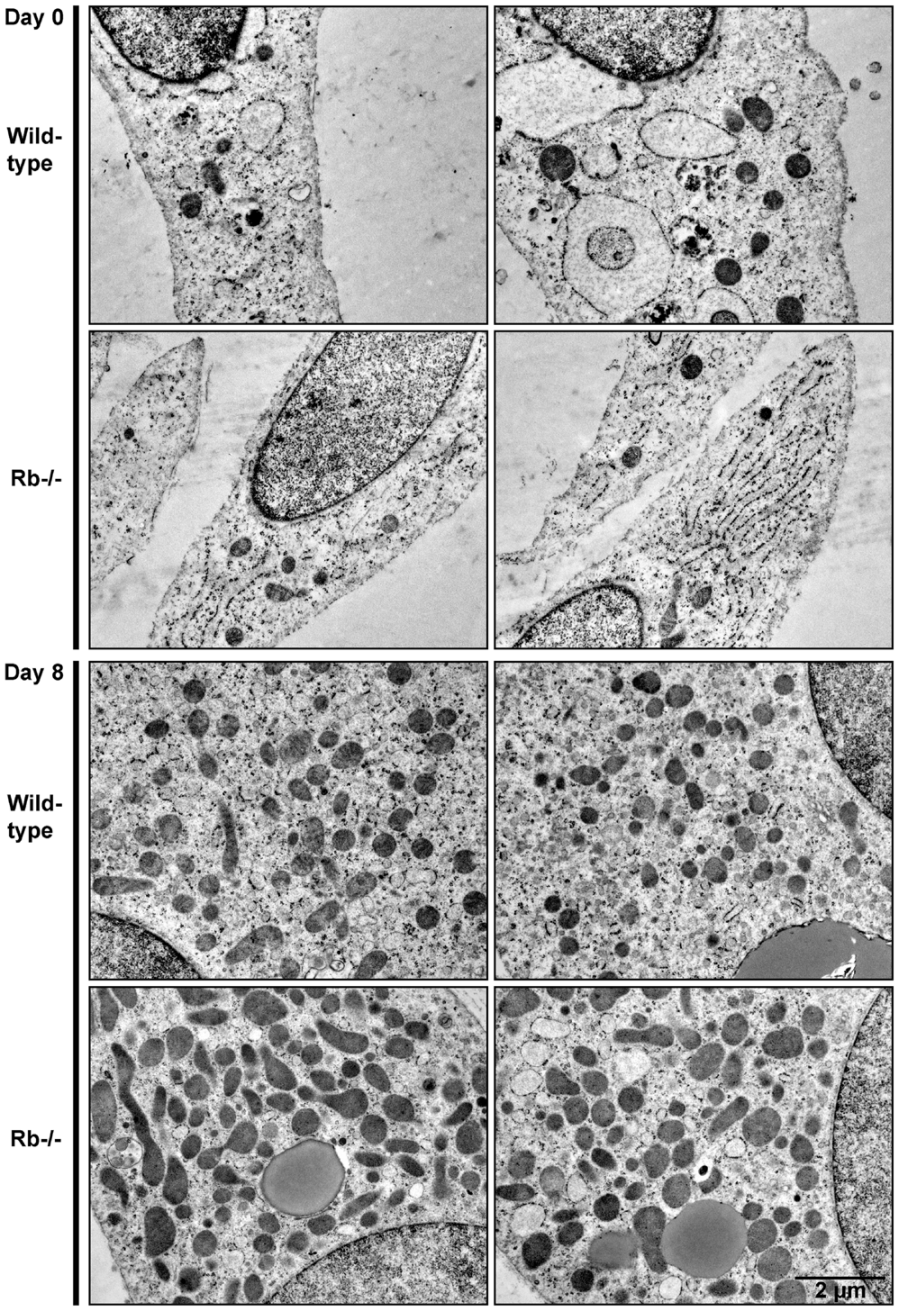
Transmission electron micrographs of cells before and after differentiation. Wild-type and Rb−/− MEFs were harvested for transmission electron microscopy at days 0 and 8 of differentiation as described in “Materials and Methods”. Two representative cells of each cell line are shown at each time point. Similar results were obtained in two independent experiments.

To obtain morphological evidence for mitochondrial biogenesis during adipocyte differentiation, wild-type and Rb−/− MEFs were investigated by electron microscopy at days 0 and 8. Confluent fibroblasts (day 0) contain relatively few mitochondria, irrespectively of Rb status (Fig. 3). Consistent with the gene expression profiles and mtDNA levels described above, mitochondrial numbers seemed to increase during differentiation of both wild-type and Rb−/− cells, and mitochondrial density appeared substantially higher in mature Rb−/− brown adipocytes (Fig. 3).

### Expression of Genes Involved in mtDNA Replication during Differentiation of White and Brown Fat Cells *In Vitro*

**Figure 4:**
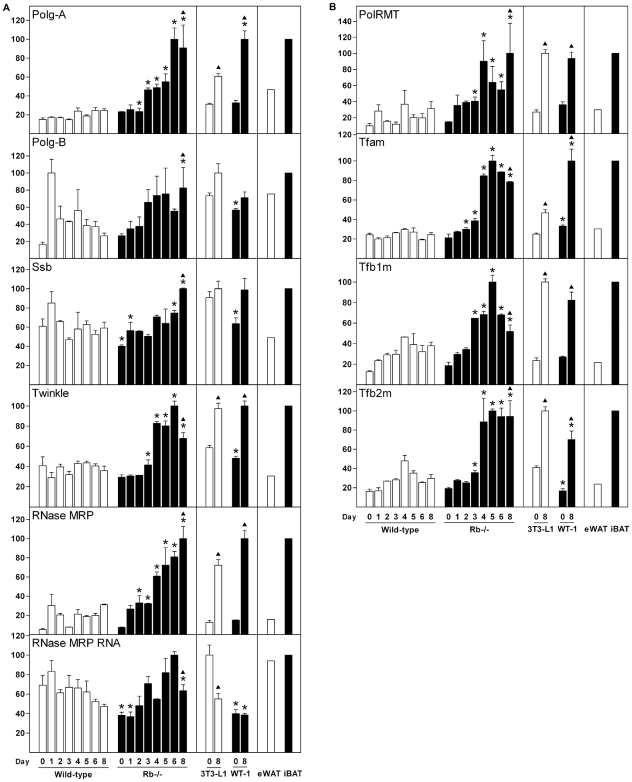
Expression of genes involved in mtDNA replication and transcription during differentiation of white and brown adipocytes. Cell lines were induced to differentiate as described in “Materials and Methods” and total RNA was harvested at the indicated days of differentiation. In addition, RNA from eWAT and iBAT was included. Expression levels were determined by RT-qPCR and relative expression levels of genes indicated in the figure determined by normalisation to the levels of TBP. In each of the three boxes for the individual genes, the mean of the normalized expression level of the sample with the highest value was set to 100. Error bars represent SEM. (**A**) Expression of genes involved in mtDNA replication. Genes measured were Polg-A, Polg-B, Ssb, Twinkle, RNase MRP and RNase MRP RNA. (**B**) Expression of genes involved in mitochondrial transcription. Genes measured were PolRMT, Tfam, Tfb1m and Tfb2m. Results from one of two independent cell culture experiments are shown. *, p<0.05 [day X in wild-type MEFs (or 3T3-L1) compared to day X in Rb−/− MEFs (or WT-1)]. ▴, p<0.05 (day 0 vs. day 8 for each of the four cell lines).

To delineate how expression of mitochondrial factors involved in replication and transcription of mtDNA was regulated, we measured their levels in the four cell lines. The functional mtDNA polymerase γ consists of a catalytic subunit (Polg-A) in complex with two copies of the accessory subunit Polg-B. Expression of the Polg subunits, Ssb, Twinkle and RNase MRP was higher in iBAT than in eWAT, whereas the RNA component of the RNase MRP (RNase MRP RNA) was expressed at similar levels in the two adipose tissues (Fig. 4A). Expression of Polg-A was relatively constant during white adipocyte differentiation of wild-type MEFs, but was progressively up-regulated during brown adipocyte differentiation of Rb−/− MEFs (Fig. 4A). Polg-A was induced 2- and 3-fold during differentiation of 3T3-L1 and WT-1 cells, respectively (Fig. 4A). The expression of Polg-B fluctuated during differentiation with a tendency towards higher expression in Rb−/− relative to wild-type cells at the later stages of differentiation. Similarly, the Ssb mRNA had a tendency towards higher expression in Rb−/− relative to wild-type cells at the later stages of differentiation, whereas the opposite was true until day 3 (Fig. 4A). In 3T3-L1 and WT-1 cells, Polg-B and Ssb were moderately induced during adipose conversion. RNase MRP was induced during fat cell differentiation of both wild-type and Rb−/− cells, but to a larger extent in the latter, whereas Twinkle was selectively induced in differentiating Rb−/− MEFs (Fig. 4A). Twinkle was moderately induced during adipose conversion of both 3T3-L1 and WT-1 cells. The expression of RNase MRP RNA in wild-type and Rb−/− cells resembled that of Ssb (Fig. 4A). Whereas RNase MRP was induced ∼5- and ∼7-fold during differentiation of 3T3-L1 and WT-1 cells, RNase MRP RNA was moderately down-regulated or constantly expressed, respectively (Fig. 4A).

### Expression of Genes Involved in Transcription of mtDNA during Differentiation of White and Brown Fat Cells *In Vitro*

PolRMT is unable to interact with promoter sequences unless assisted by Tfam and either Tfb1m or Tfb2m [7], [9]. mTERF1, and potentially also mTERF2-4, is involved in termination of mtDNA transcripts, but has also recently been suggested to promote transcription initiation. PolRMT was induced early during adipose conversion of wild-type and Rb−/− MEFs, with levels beginning to increase at day 1, and levels in Rb−/− cells exceeding that of wild-type cells from day 2 (Fig. 4B). Similarly, PolRMT was induced during adipose conversion of both 3T3-L1 and WT-1 cells. Expression of Tfam and Tfb2m was relatively constant in differentiating wild-type cells, whereas both increased ∼4-fold in Rb−/− cells (Fig. 4B). Tfb1m expression was induced already at day 1 and continued to increase until day 4 or 5, after which expression was stable (wild-type cells) or decreased (Rb−/− cells) (Fig. 4B). The level of Tfb1m was moderately higher after day 2 in Rb−/− compared with wild-type cells. Tfam and Tfb2m were both induced ∼2-fold during differentiation of 3T3-L1 and ∼3-fold during differentiation of WT-1 cells (Fig. 4B). In contrast, Tfb1m was induced ∼4-fold and ∼3-fold during differentiation of 3T3-L1 and WT-1 cells, respectively. The mitochondrial ribosomal protein L12 (Mrpl12) has been demonstrated to enhance mtDNA transcription via physical interaction with PolRMT [43]. Mrpl12 was induced ∼5-fold and ∼20-fold during differentiation of wild-type and Rb−/− MEFs, respectively, with expression beginning to increase earlier in the latter (Fig. S3). Expression of Mrpl12 was strongly induced during adipose conversion of both 3T3-L1 and WT-1 cells. Fluctuations of the mTERF1 and mTERF3 mRNAs were observed during differentiation of MEFs, with levels being moderately higher in Rb−/− relative to wild-type mature adipocytes (Fig. S3). mTERF1 was slightly down-regulated during differentiation of 3T3-L1 cells, whereas it was moderately induced during WT-1 adipogenesis (Fig. S3). mTERF2 was transiently down-regulated in differentiating MEFs and expression levels were ∼2-fold higher in Rb−/− relative to wild-type cells on most days (Fig. S3). During differentiation of MEFs, mTERF4 was selectively induced in Rb−/− cells. mTERF2, mTERF3 and mTERF4 were induced 1.5-3-fold during differentiation of both 3T3-L1 and WT-1 cells (Fig. S3). PolRMT, Tfam, Tfb1m, Tfb2m, Mrpl12, mTERF2, mTERF3 and mTERF4 were enriched in iBAT relative to eWAT, whereas mTERF1 was expressed at similar levels (Fig. 4B and S3).

### Expression of the PGC-1 Co-Activator Family and the RIP140 Co-Repressor during Differentiation of White and Brown Fat Cells *In Vitro*

**Figure 5:**
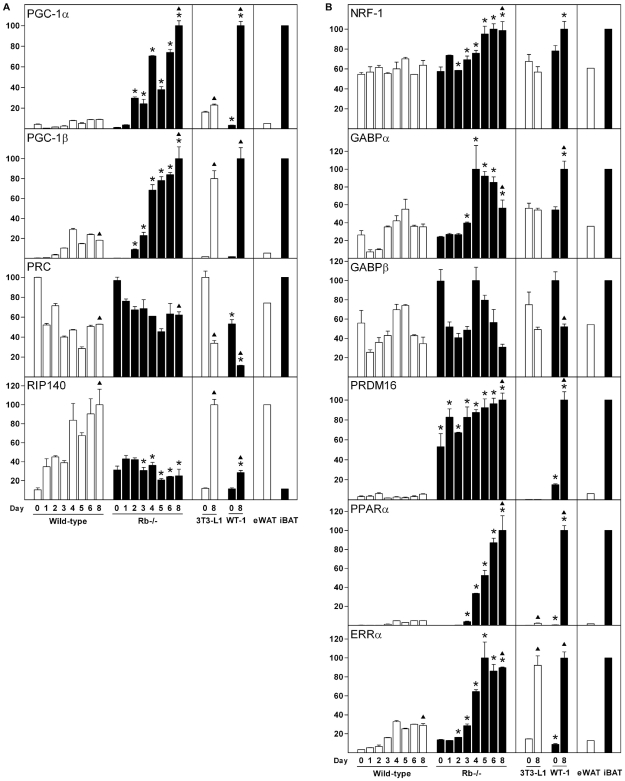
Expression of nuclear co-regulators and DNA-binding transcription factors linked to mitochondrial biogenesis and function during differentiation of white and brown fat cells. Cell lines were induced to differentiate as described in “Materials and Methods” and total RNA was harvested at the indicated days of differentiation. In addition, RNA from eWAT and iBAT was included. Expression levels were determined by RT-qPCR and relative expression levels of genes indicated in the figure determined by normalisation to the levels of TBP. In each of the three boxes for the individual genes, the mean of the normalized expression level of the sample with the highest value was set to 100. Error bars represent SEM. (**A**) Expression of PGC-1 family members and RIP140. (**B**) Expression of the nuclear DNA-binding transcription factors NRF-1, GABPα, GABPβ, PRDM16, PPARα and ERRα. Results from one of two independent cell culture experiments are shown. *, p<0.05 [day X in wild-type MEFs (or 3T3-L1) compared to day X in Rb−/− MEFs (or WT-1)]. ▴, p<0.05 (day 0 vs. day 8 for each of the four cell lines).

The PGC-1 family comprises, in addition to PGC-1α and PGC-1β, also the PGC-1-related co-activator (PRC) [44]. PGC-1α and PGC-1β are expressed at much higher levels in iBAT relative to eWAT, whereas PRC is present in equal amounts in eWAT and iBAT (Fig. 5A) [16], [44], [45]. PGC-1α was induced ∼70-fold and ∼30-fold during differentiation of Rb−/− cells and WT-1 cells, respectively, whereas expression was barely induced in wild-type MEFs and 3T3-L1 cells (Fig. 5A). Of notice, PGC-1α was induced as early as 24 h after induction of differentiation of Rb−/− MEFs, whereas PGC-1α was transiently down-regulated at the same time in wild-type MEFs. PGC-1β was strongly induced during differentiation of all four cell models used, but was induced to the lowest level in wild-type MEFs (Fig. 5A). Contrary to PGC-1α and PGC-1β, expression of PRC was gradually down-regulated during differentiation of wild-type and Rb−/− cells (Fig. 5A). Curiously, PRC was expressed at the highest level in exponentially growing MEFs, at which point PGC-1α and PGC-1β were barely detectable (data not shown). PRC was also down-regulated during differentiation of 3T3-L1 and WT-1 cells (Fig. 5A). Disruption of the co-repressor RIP140 in mice results in increased expression of genes involved in energy dissipation in white adipose tissue and cultured fat cells, suggesting that RIP140 is normally promoting energy conservation. As previously reported [46], [47], expression of RIP140 mRNA was increased 8-fold during white adipocyte differentiation of wild-type MEFs and 3T3-L1 cells (Fig. 5A). In contrast, expression of RIP140 was relatively constant during brown adipose conversion of Rb−/− MEFs and ∼4-fold lower than in wild-type cells in the adipose state (Fig. 5A). In WT-1 cells, RIP140 was induced ∼2-fold during differentiation.

### Expression of Nuclear DNA-Binding Transcription Factors Linked to Mitochondrial Biogenesis and Function during Differentiation of White and Brown Fat Cells *In Vitro*

Expression of NRF-1 was relatively constant during white adipocyte differentiation of wild-type MEFs and was induced 1.7-fold at late stages of brown adipogenesis of Rb−/− MEFs (Fig. 5B). In 3T3-L1 and WT-1 cells, NRF-1 was moderately down- and up-regulated, respectively. NRF-1 was expressed at moderately (1.6-fold) higher levels in iBAT relative to eWAT. GABPα and GABPβ displayed a similar expression pattern during differentiation of white and brown fat cells, with a transient down-regulation at days 1 and 2 (except for GABPα in Rb−/− cells) as well as a second down-regulation at the terminal stages of differentiation (days 6 and 8) (Fig. 5B). In addition, whereas GABPβ was expressed at similar levels in wild-type and Rb−/− cells during differentiation, GABPα expression was moderately higher on most days in Rb−/− cells. GABPα was induced nearly 2-fold during brown adipose conversion of WT-1 cells, whereas its expression was unchanged from day 0 to day 8 in 3T3-L1 cells. In differentiating 3T3-L1 and WT-1 cells, GABPβ was moderately down-regulated. In eWAT and iBAT, GABPα and GABPβ were expressed at ∼2-fold higher levels in the latter (Fig. 5B). Of interest, PRDM16 was expressed at dramatically higher levels in Rb−/− MEFs compared to wild-type MEFs (∼15-fold) and in WT-1 pre-adipocytes compared to 3T3-L1 pre-adipocytes (∼50-fold) even before differentiation was initiated (day 0). In addition, expression of PRDM16 was increased further during differentiation of Rb−/− and WT-1 cells. PPARα was induced during adipose conversion of all cell types, but were induced to much higher levels in Rb−/− and WT-1 adipocytes compared with wild-type and 3T3-L1 fat cells (Fig. 5B). Expression of ERRα was induced ∼6-fold during differentiation of wild-type and Rb−/− MEFs, but ERRα levels were substantially higher in mature Rb−/− relative to wild-type adipocytes. In 3T3-L1 and WT-1 cells, ERRα was induced ∼6- and ∼12-fold during differentiation (Fig. 5B). Both PPARα and ERRα were enriched in iBAT relative to eWAT, consistent with previous findings [48]–[50]. As SHP-deficiency has been shown to increase expression of PGC-1α and UCP1 as well as mitochondrial biogenesis [22], we also measured the expression of SHP. Consistent with previously reported data [51] (www.nursa.org/10.1621/datasets.01006; www.nursa.org/10.1621/datasets.02001), we failed to reproducibly detect expression of SHP in eWAT and iBAT as well as in the cell lines used in this study (data not shown).

### Gene Expression in Brown Adipose Tissue Fractions and Adipose Tissues of Cold-Exposed Mice

Based on the expression profiles described above, we decided to explore the regulation of a selection of genes in two *in vivo* situations: in fractionated BAT as well as in inguinal WAT (iWAT) and iBAT from mice exposed to cold for 1 or 3 days. Comparison of the pre-adipocyte-containing stromal-vascular fraction and adipose fraction (SVF and AF, respectively) of BAT provides a measure of the regulation of genes during brown adipose conversion *in vivo*. Cold exposure of mice results in activation of existing BAT (short-term effect) as well as recruitment of new brown fat cells (long-term effect), whereas long-term cold exposure causes a conversion of some WAT depots, such as iWAT but not eWAT, into BAT-like adipose tissue containing UCP1-positive fat cells [28], [29], [39], [52], [53].

**Figure 6:**
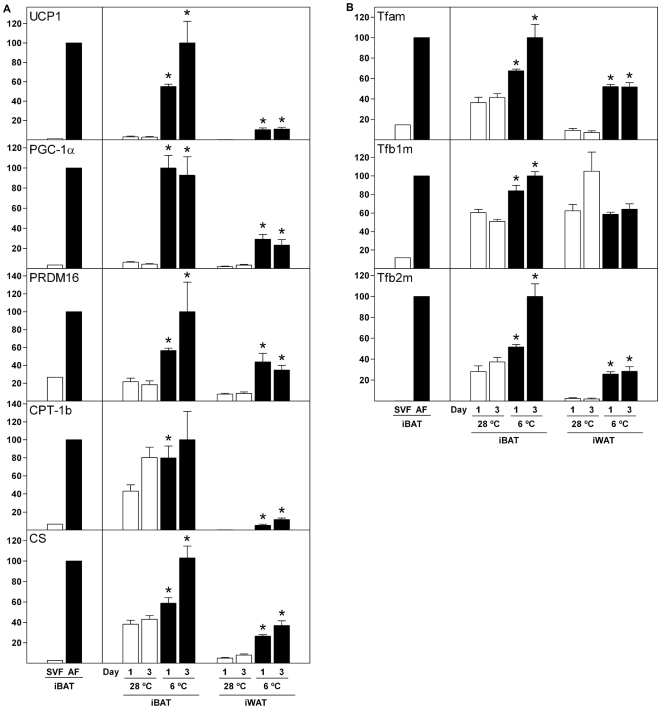
Expression of selected genes in brown adipose tissue fractions and adipose tissues from cold-challenged mice. RNA from BAT fractions (pool from 12 mice) and adipose tissues from cold-challenged mice (4 animals in each group) was analysed by RT-qPCR. Data for SVF and AF samples from BAT are shown in the left box, whereas data for samples from iBAT and iWAT of cold-challenged mice are shown in the right box. Expression levels in BAT fractions were normalised to the expression of 18S rRNA. Expression levels in adipose tissue from cold-challenged mice were normalised to the expression of TBP. In each of the two boxes for the individual genes, the mean of the normalized expression level of the sample with the highest value was set to 100. Error bars represent SEM. (**A**) Expression of UCP1, PGC-1α, PRDM16, CPT-1b and CS. (**B**) Expression of Tfam, Tfb1m and Tfb2m. *, p<0.05 [day X at 28°C in iBAT (or iWAT) compared to day X at 6°C in iBAT (or iWAT)].

Proper separation of BAT SVF from AF was validated by the expression of PPARγ2, adiponectin, UCP1, PGC-1α, PRDM16 and CPT-1b (Fig. S4 and 6A). Consistent with the CS expression patterns in Fig. 1B and the expectation of mitochondrial biogenesis occurring during brown fat cell differentiation *in vivo*, CS mRNA was more than 30-fold higher in AF compared with SVF (Fig. 6A). In mice exposed to 6°C for 1 or 3 days [28], [29], UCP1 and PGC-1α were massively induced in iBAT and iWAT compared with mice maintained at 28°C, with expression reaching 4–6-fold higher levels in iBAT compared to iWAT (Fig. 6A). In addition, PRDM16 was cold-inducible in both iBAT and iWAT. CPT-1b was expressed at low levels in iWAT at 28°C, but was induced upon exposure to cold. Expression of CPT-1b was not significantly affected by cold in iBAT, but was expressed at 6–10-fold higher levels than in iWAT from cold-exposed mice (Fig. 6A). CS was expressed at higher levels in iBAT than in iWAT in mice housed at 28°C, and cold exposure caused a ∼2-fold and ∼5-fold induction of CS in iBAT and iWAT, respectively (Fig. 6A). As we were intrigued by the expression patterns of Tfam, Tfb1m and Tfb2m described above, we measured their expression in fractionated BAT as well as in adipose tissues of cold-challenged mice. Tfam and Tfb1m were expressed at 7–8-fold higher levels in AF relative to SVF. Tfb2m was barely detectable in SVF, but was highly expressed in AF (Fig. 6B). Tfam was induced ∼2-fold in iBAT and ∼6-fold and iWAT in response to cold. Upon cold exposure, expression of Tfb1m was moderately induced in iBAT but not in iWAT (Fig. 6B). In contrast, Tfb2m was induced ∼10-fold in iWAT after a cold challenge, whereas its expression increased 2–3-fold in iBAT (Fig. 6B).

### Forced Expression of Tfam or Knockdown of PRDM16 Influences Mitochondrial Biogenesis in Brown Adipocytes and Precursor Cells

**Figure 7:**
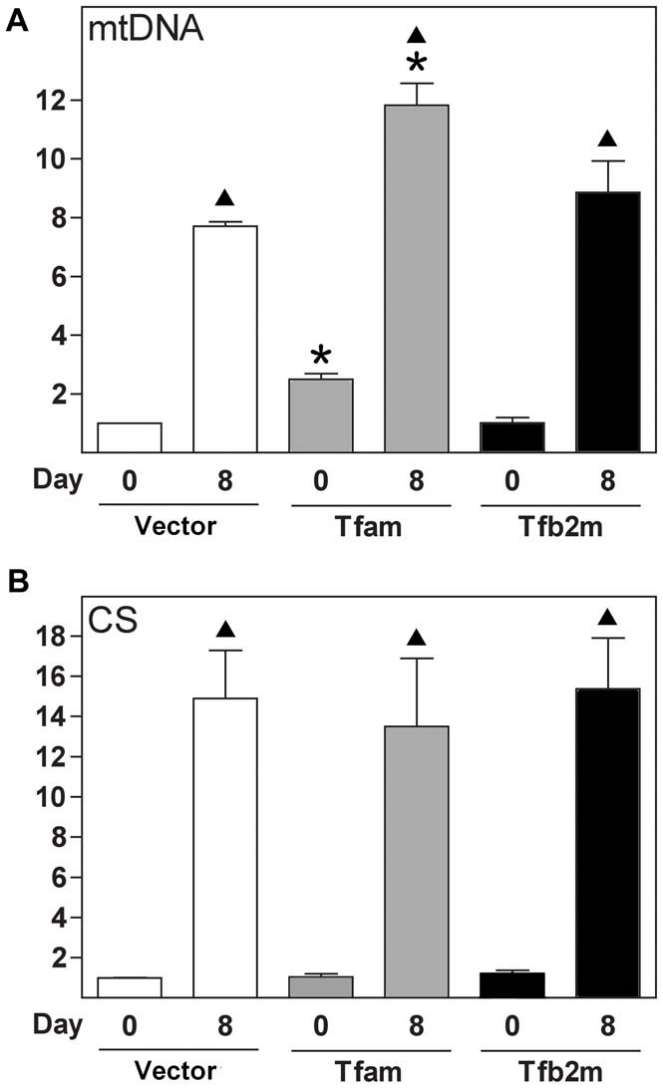
Effect of forced expression of Tfam and Tfb2m on mtDNA replication and citrate synthase expression. Rb−/− MEFs were transduced with an empty retrovirus (designated “Vector”) or retroviral vectors encoding Tfam or Tfb2m. Total DNA and RNA were harvested at days 0 and 8. (**A**) Relative mtDNA copy number was determined by qPCR using primer sets specific for mtDNA (COX II) and nDNA (RIP140) and relative mtDNA levels calculated by normalising signals from COX II to those of RIP140. (**B**) Expression levels were determined by RT-qPCR and the relative expression level of CS determined by normalisation to the level of TBP. The results presented are the means from three independent experiments. The Vector sample on day 0 was set to 1 in each experiment. Error bars represent SEM. *, p<0.05 (day X in Vector compared to day X in Tfam or Tfb2m). ▴, p<0.05 (day 0 vs. day 8 for Vector, Tfam or Tfb2m).

Next, we determined the relevance of Tfam, Tfb2m and PRDM16 for the observations relating to mitochondrial biogenesis described above. We used retroviral vectors for delivery into Rb−/− MEFs of Tfam cDNA, Tfb2m cDNA and short-hairpin RNA (shRNA) against PRDM16. In the case of transduction with Tfam- or Tfb2m-encoding retrovirus, we harvested cells prior to induction of differentiation (day 0) to determine if mitochondrial biogenesis was affected independent of adipose conversion. Overexpression of Tfam and Tfb2m was confirmed by RT-qPCR (data not shown). Tfam overexpression resulted in 2- and 1.5-fold increases in the relative levels of mtDNA at days 0 and 8 compared with cells transduced with control retrovirus (Vector cells), whereas overexpression of Tfb2m was without effect (Fig. 7A). However, forced expression of Tfam did not result in increased expression of CS mRNA (Fig. 7B). These data suggest that increasing Tfam levels are sufficient to induce mtDNA replication in MEFs, even before differentiation is induced.

**Figure 8:**
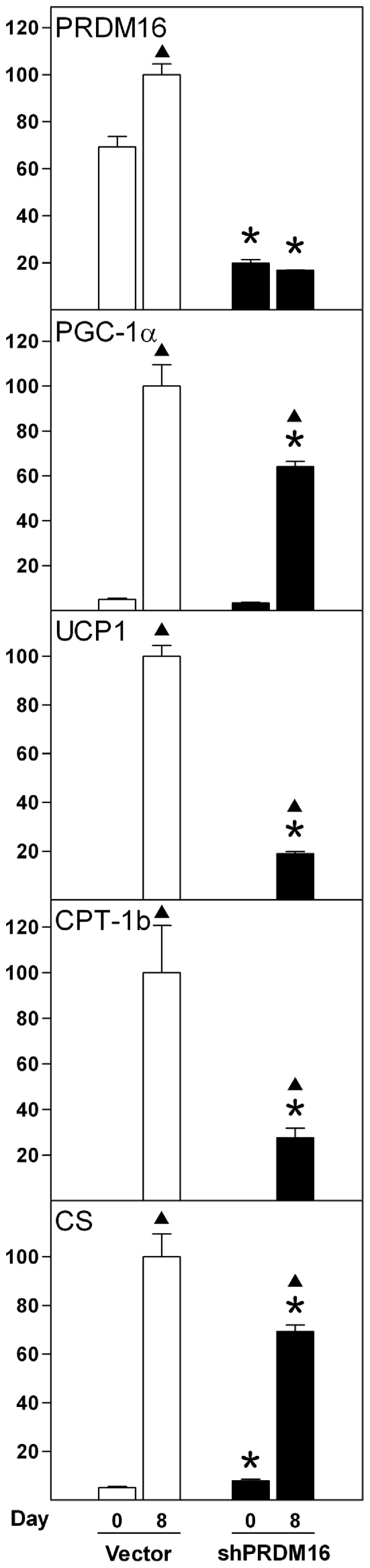
Effect of silencing of PRDM16 expression on expression levels of mitochondrial marker genes. Rb−/− MEFs were transduced with pSUPER.retro.neo or pSUPER.retro.neo-PRDM16 virus (designated “Vector” and “shPRDM16”, respectively), selected, replated and induced to differentiate as described in “Materials and Methods” and total RNA was harvested at days 0 and 8 of differentiation. Expression levels were determined by RT-qPCR and relative expression levels of genes indicated in the figure determined by normalisation to the levels of TBP. In each of the boxes for the individual genes, the mean of the normalized expression level of the sample with the highest value was set to 100. Error bars represent SEM. Shown is the expression of PRDM16, PGC-1α, UCP1, CPT-1b and CS. A representative of three independent experiments is shown. *, p<0.05 (day X in Vector compared to day X in shPRDM16). ▴, p<0.05 (day 0 vs. day 8 for Vector or shPRDM16).

As described above, PRDM16 is expressed at remarkably higher levels in Rb−/− relative to wild-type MEFs and in WT-1 relative to 3T3-L1 pre-adipocytes, both in the undifferentiated and fully differentiated state (Fig. 5B). To address whether the differential expression of PRDM16 in MEFs was of relevance for the observed differences in mitochondria-related gene expression and biogenesis, we silenced PRDM16 expression in Rb−/− MEFs by retroviral delivery of shRNA. Expression of the shRNA reduced PRDM16 mRNA levels to 30% at day 0 and 17% at day 8 relative to Vector cells (Fig. 8). Of notice, silencing of PRDM16 caused a 1.7-fold induction of myogenin expression at day 0, an induction that did not reach statistical significance (Fig. S5A). Silencing of PRDM16 did not inhibit differentiation *per se*, as measured by expression of PPARγ2, adiponectin and FABP4 at day 8 (Fig. S5A). Consistent with previous finding [4], [19], [25], the knockdown caused a reduction of UCP1 and CPT-1b levels of 5- and 3-fold, respectively, whereas PGC-1α was reduced by 40% at day 8 (Fig. 8). Induction of CS expression during differentiation was blunted by 30% in PRDM16 knockdown cells compared to Vector cells (Fig. 8). In addition, expression of GABPα, PPARα and Cidea was lower in cells expressing shRNA against PRDM16 (Fig. S5B and data not shown). In contrast, no effect was observed on expression of Tfam, Tfb1m, Tfb2m, PGC-1β, Polg-A and PolRMT (Fig. S5B and data not shown). Together, these data support a powerful role of PRDM16 in the formation of functional brown adipocytes, including mitochondrial biogenesis and expression of UCP1.

## Discussion

The capacity of brown fat cells to dissipate significant amounts of energy as heat requires a high mitochondrial density and a mechanism of uncoupling oxidative phosphorylation. The former requirement is met by a mitochondrial volume fraction of BAT of approximately 30% [54], and the latter by the unique presence of UCP1 at high levels in the mitochondrial inner membrane. In the present report, we provide a detailed analysis of gene expression linked to replication and transcription of mtDNA as well as expression profiles of nuclear transcription regulators of mitochondrial function during white and brown fat cell differentiation *in vitro* and in adipose tissues from cold-challenged mice.

Adipocyte differentiation is associated with mitochondrial biogenesis [55]. Corroborating this, we measured a substantial induction of CS expression and mtDNA copy number during differentiation of both white and brown adipocytes, with induction being particularly pronounced during brown adipogenesis (Fig. 1B). Several genes encoding transcription regulators of mitochondrial function were differently regulated during white and brown fat cell differentiation *in vitro*. Of those acting in the nucleus, PRDM16, NRF-1, GABPα, PPARα and PGC-1α were expressed or induced preferentially during brown adipose conversion, whereas the co-repressor RIP140 was induced primarily during white fat cell differentiation. Tfam and Tfb2m were selectively induced in differentiating Rb−/− MEFs compared to wild-type MEFs and elicited a more pronounced induction during differentiation of WT-1 relative to 3T3-L1 pre-adipocytes.

Tfam is a key regulator of mtDNA transcription, mtDNA copy number and mitochondrial biogenesis [13], [56], [57]. Moreover, Tfb2m promotes mtDNA transcription more efficiently than Tfb1m [58]–[60]. As mentioned above, expression of Tfam, Tfb1m and Tfb2m were enriched in iBAT relative to eWAT and their levels were substantially higher in AF compared to SVF of BAT. Moreover, all three were induced during brown adipocyte differentiation *in vitro* and induction of particularly Tfam and Tfb2m was more pronounced during brown relative to white adipogenesis. This is consistent with the larger increase in mtDNA copy number and increased mitochondrial biogenesis during the differentiation of brown fat cells *in vitro*. In cold-challenged mice, the three mitochondrial transcription factors were moderately induced in iBAT, whereas Tfam and Tfb2m were strongly induced in iWAT. We show that forced expression of Tfam but not Tfb2m in adipocyte precursor cells results in increased mtDNA levels in both the undifferentiated (2-fold) and differentiated state (1.5-fold), suggesting that Tfam promotes mitochondriogenesis in these cells. The doubling of mtDNA on day 0 by Tfam overexpression is relatively minor compared to the 7-fold increase observed during differentiation of Rb−/− cells (Fig. 2B). This suggests that Tfam alone is unable to drive the level of mitochondrial biogenesis observed during brown adipose conversion and that additional factors are required. Such additional factors might be any of the nuclear transcription factors involved in mitochondrial function or any of the mitochondrial transcription or replication factors. Nevertheless, our expression profiles support a role of Tfam and Tfb2m in controlling the differential mtDNA replication and mitochondrial biogenesis during white and brown adipocyte differentiation. Finally, the induction of Tfam and Tfb2m in iWAT following cold exposure suggests their involvement in the mitochondriogenesis taking place (as indicated by the induction of CS) during the transformation to a BAT-like depot. Decisive evidence for a functional role of Tfam, Tfb1m and Tfb2m in mitochondrial biogenesis and function in adipose tissue awaits the generation of tissue-specific knockout mice.

The identification of the causative signal that triggers mitochondrial biogenesis and UCP1 expression during brown adipose conversion is key to establishing the molecular background for the differential metabolic functions of white and brown fat. PGC-1α and PGC-1β are crucial in brown adipogenesis, being necessary for both mitochondrial biogenesis and UCP1 expression *in vitro* and *in vivo*. However, these effects require that both factors are absent simultaneously, as absence of either PGC-1α or PGC-1β has little effect [14], [15]. Therefore, PGC-1α or PGC-1β is unlikely to be the triggering factor that causes functional brown adipocyte differentiation. We find that PGC-1α is not enriched in precursor cells (day 0) destined to become brown fat cells (Rb−/− MEFs and WT-1 pre-adipocytes) relative to those destined to become white adipocytes (wild-type MEFs and 3T3-L1 pre-adipocytes). However, PGC-1α is induced as early as day 1 in Rb−/− cells, whereas PGC-1β is induced one day later. Overall, our expression profiles support an important role of PGC-1α and PGC-1β in brown adipocyte differentiation and function, but not as triggering factors. Contrary, PRDM16 is expressed at dramatically higher levels in Rb−/− relative to wild-type MEFs and in WT-1 relative to 3T3-L1 pre-adipocytes prior to differentiation (Fig. 4B). In exponentially growing cultures of wild-type MEFs, we failed to detect expression of PRDM16, whereas it was easily detectable in proliferating Rb−/− MEFs (data not shown). PRDM16 is strongly enriched in iBAT relative to eWAT and is expressed at higher levels in brown AF compared to SVF (Fig. 6A) [19], but it has not been reported whether PRDM16 is enriched in SVF from BAT relative to SVF from WAT. In cold-challenged mice, PRDM16 expression is induced in both iBAT and iWAT (Fig. 6A). Based on the powerful effect of PRDM16 on brown adipose conversion of white pre-adipocytes and myoblasts as well as its reported expression patterns, PRDM16 is a prime candidate as priming and triggering factor for brown adipocyte differentiation. Consistent with previous reports [4], [19], [25], silencing of PRDM16 in brown adipocyte precursor cells (in this case Rb−/− MEFs) blunted the induction of typical brown fat marker genes, like UCP1, CPT-1b and PGC-1α (Fig. 8). Moreover, induction of CS expression was reduced in PRDM16 knockdown cells, suggesting that mitochondrial biogenesis was diminished. However, mitochondrial transcription and replication factors were not substantially affected. The reason for the relatively minor effect of PRDM16 knockdown on CS expression and the lack of effect on induction of mitochondrial factors might be ascribed to a knockdown of only ∼3–6-fold, which means that the remaining amount of PRDM16 mRNA still exceeds the amount present in the corresponding wild-type cells (see Fig. 5B). It will be relevant to clarify how expression of PRDM16 is regulated, as little is known about signalling pathways and transcription factors impacting on the PRDM16 promoter. However, the observation that PRDM16 is enriched in cells with the capacity to differentiate to brown adipocytes relative to comparable cells with the capacity to become white fat cells, even before adipogenesis is induced, suggests that the PRDM16 gene is subject to differential epigenetic regulation in white and brown pre-adipocytes.

Recent findings demonstrate the existence of active BAT in a subset of adult humans [61]–[66]. Based on the anti-obesity function of BAT in rodents, a better understanding of the proliferative and thermogenic potential of this tissue is of significant interest. As mitochondria are important for BAT function, detailed information on processes leading to mitochondrial biogenesis in adipocytes is of potential relevance for the development of future anti-obesity regimens.

Here we have identified a highly dynamic pattern of expression of genes involved in replication and transcription of mtDNA as well as of nuclear transcription factors regulating mitochondrial function during white and brown adipocyte differentiation. Specific or selective induction of a number of these was observed during brown adipose conversion, including PGC-1α, Tfam and Tfb2m. PRDM16 was expressed at higher levels in brown compared to white adipocyte precursor cells. We provide evidence that modulation of Tfam and PRDM16 levels affect mitochondrial DNA replication, gene expression and biogenesis during fat cell differentiation. In summary, the molecular machinery controlling mitochondrial biogenesis and function is differentially regulated during white and brown adipocyte differentiation, and our data are consistent with a key role of PRDM16 in priming and triggering brown adipogenesis.

## Supporting Information

Figure S1 Expression of general adipose markers during differentiation of white and brown fat cells. Cell lines were induced to differentiate as described in “Materials and Methods” and total RNA was harvested at the indicated days of differentiation. In addition, RNA from eWAT and iBAT was included. Expression levels were determined by RT-qPCR and relative expression levels of genes indicated in the figure determined by normalisation to the levels of TBP. In each of the three boxes for the individual genes, the mean of the normalized expression level of the sample with the highest value was set to 100. Error bars represent SEM. Genes measured were PPARÎ^3^2, C/EBPÎ±, FABP4 and adiponectin. Results from one of two independent cell culture experiments are shown. *, p<0.05 [day X in wild-type MEFs (or 3T3-L1) compared to day X in Rb−/− MEFs (or WT-1)]. Δ, p<0.05 (day 0 vs. day 8 for each of the four cell lines).
(0.87 MB TIF)

Figure S2 Expression of myogenin in white and brown adipocyte precursor cells. Total RNA was harvested at day 0 and relative expression of myogenin was determined by RT-qPCR by normalisation to TBP. In each of the two boxes, the mean of the normalized expression level of the sample with the highest value was set to 100. Error bars represent SEM. Results from one of two independent experiments are shown. *, p<0.05 [wild-type MEFs (or 3T3-L1) compared to Rb−/− MEFs (or WT-1)].
(0.16 MB TIF)

Figure S3 Expression of genes involved in mitochondrial transcription during differentiation of white and brown adipocytes. Cell lines were induced to differentiate as described in “Materials and Methods” and total RNA was harvested at the indicated days of differentiation. In addition, RNA from eWAT and iBAT was included. Expression levels were determined by RT-qPCR and relative expression levels of genes indicated in the figure determined by normalisation to the levels of TBP. In each of the three boxes for the individual genes, the mean of the normalized expression level of the sample with the highest value was set to 100. Error bars represent SEM. Genes measured were Mrpl12 and mTERF1-4. Results from one of two independent cell culture experiments are shown. *, p<0.05 [day X in wild-type MEFs (or 3T3-L1) compared to day X in Rb−/− MEFs (or WT-1)]. Δ, p<0.05 (day 0 vs. day 8 for each of the four cell lines).
(0.95 MB TIF)

Figure S4 Expression of PPARγ2 and adiponectin in stromal-vascular and adipose fractions of brown adipose tissue. RNA from BAT fractions (pool from 12 mice) was analysed for the expression of PPARγ2 and adiponectin by RT-qPCR. Expression levels were normalised to the expression of 18S rRNA. The highest value of the mean of the normalized expression of PPARγ2 or adiponectin was set to 100.
(0.07 MB TIF)

Figure S5 Expression of selected transcription factors after silencing of PRDM16 expression. Rb−/− MEFs were transduced with pSUPER.retro.neo or pSUPER.retro.neo-PRDM16 virus (designated “Vector” and “shPRDM16”, respectively), selected, replated and induced to differentiate as described in “Materials and Methods” and total RNA was harvested at days 0 and 8 of differentiation. Expression levels were determined by RT-qPCR and relative expression levels of genes indicated in the figure determined by normalisation to the levels of TBP. In each of the boxes for the individual genes, the mean of the normalized expression level of the sample with the highest value was set to 100. Error bars represent SEM. (A) Expression of PPARÎ^3^2, adiponectin, FABP4 and myogenin. (B) Expression of Tfam, Tfb1m, Tfb2m and GABPÎ±. Similar results were obtained in three independent experiments. Similar results were obtained in three independent experiments. *, p<0.05 (day X in Vector compared to day X in shPRDM16). Δ, p<0.05 (day 0 vs. day 8 for Vector or shPRDM16).
(0.49 MB TIF)

Table S1 Primers used for quantitative PCR
(0.09 MB DOC)

Table S2 Normalised expression levels of analysed genes. To allow comparison of expression levels across cell lines and tissues, relevant samples normalised to TBP (gene of interest/TBP) are listed. Expression levels for BAT fractions are not provided, as normalisation in this case was to 18S rRNA.
(0.38 MB DOC)
