# Supplementary figures and images for: Dynamic Regulation of Genes Involved in Mitochondrial DNA Replication and Transcription during Mouse Brown Fat Cell Differentiation and Recruitment
Source: PLoS One. 2009 Dec 24;4(12):e8458. doi: 10.1371/journal.pone.0008458 (PMC2809086; doi:10.1371/journal.pone.0008458)

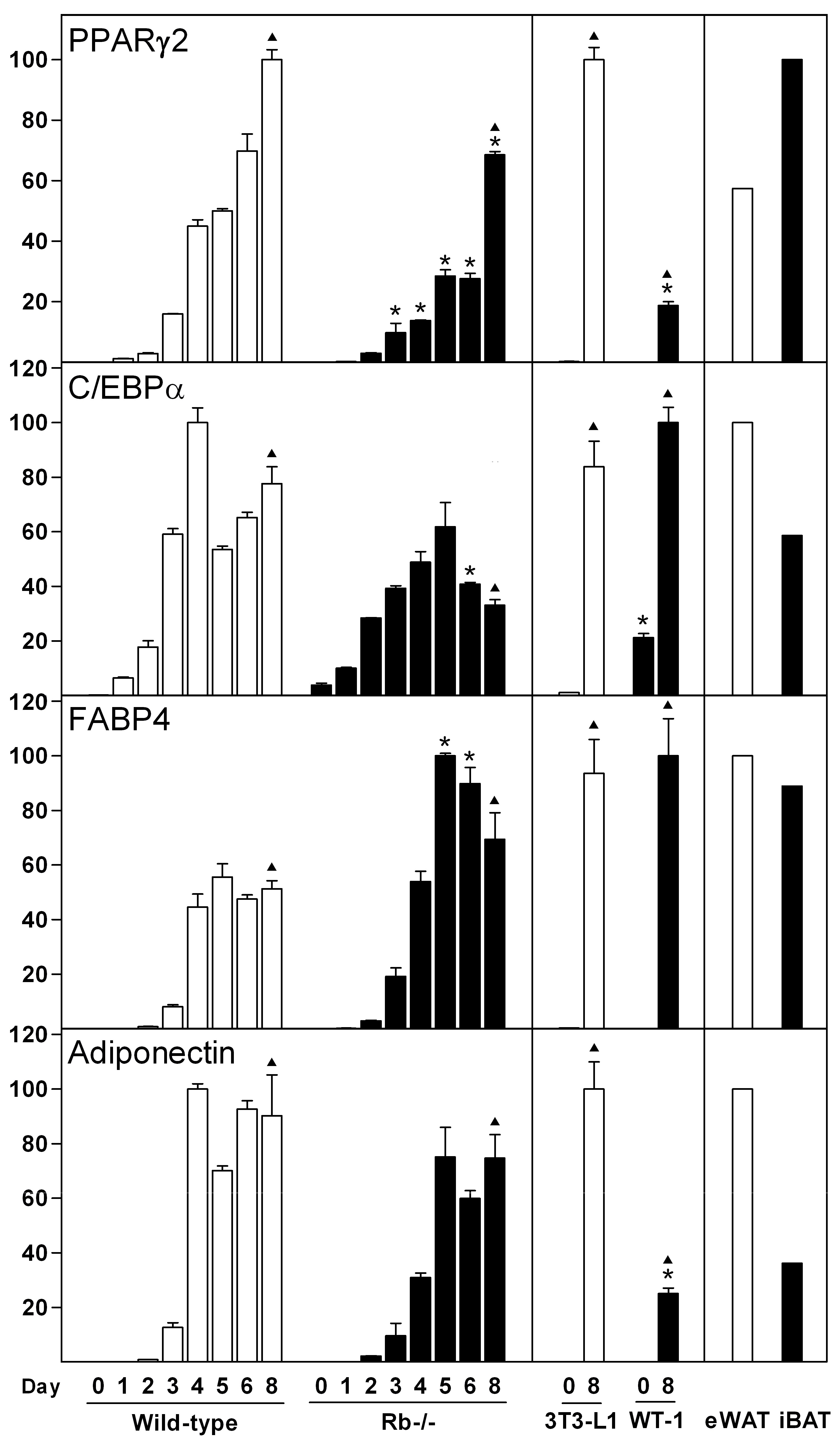

Supplement: Figure S1 — Expression of general adipose markers during differentiation of white and brown fat cells. Cell lines were induced to differentiate as described in “Materials and Methods” and total RNA was harvested at the indicated days of differentiation. In addition, RNA from eWAT and iBAT was included. Expression levels were determined by RT-qPCR and relative expression levels of genes indicated in the figure determined by normalisation to the levels of TBP. In each of the three boxes for the individual genes, the mean of the normalized expression level of the sample with the highest value was set to 100. Error bars represent SEM. Genes measured were PPARÎ32, C/EBPÎ±, FABP4 and adiponectin. Results from one of two independent cell culture experiments are shown. *, p<0.05 [day X in wild-type MEFs (or 3T3-L1) compared to day X in Rb−/− MEFs (or WT-1)]. Δ, p<0.05 (day 0 vs. day 8 for each of the four cell lines). (0.87 MB TIF) [file pone.0008458.s001.tif]

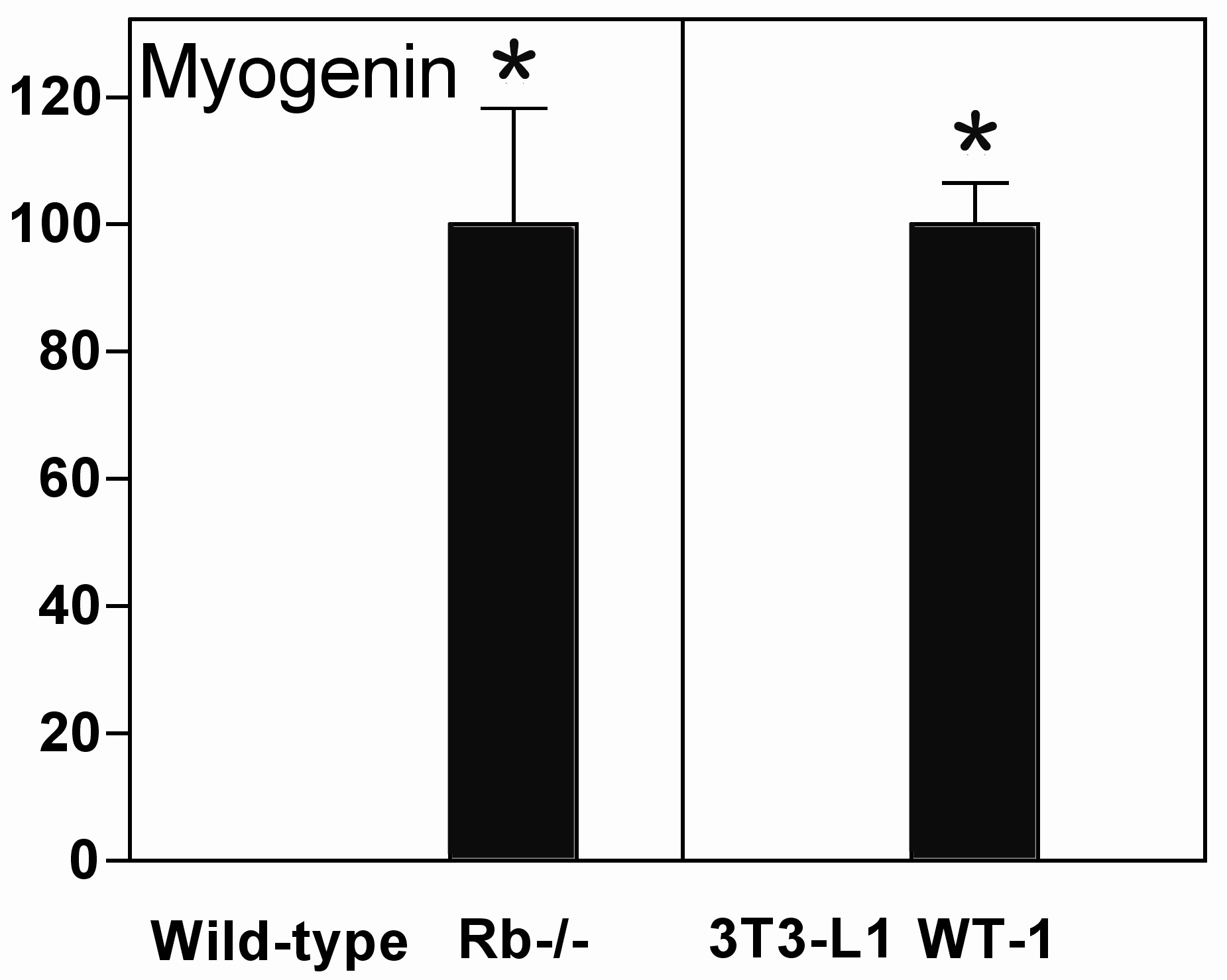

Supplement: Figure S2 — Expression of myogenin in white and brown adipocyte precursor cells. Total RNA was harvested at day 0 and relative expression of myogenin was determined by RT-qPCR by normalisation to TBP. In each of the two boxes, the mean of the normalized expression level of the sample with the highest value was set to 100. Error bars represent SEM. Results from one of two independent experiments are shown. *, p<0.05 [wild-type MEFs (or 3T3-L1) compared to Rb−/− MEFs (or WT-1)]. (0.16 MB TIF) [file pone.0008458.s002.tif]

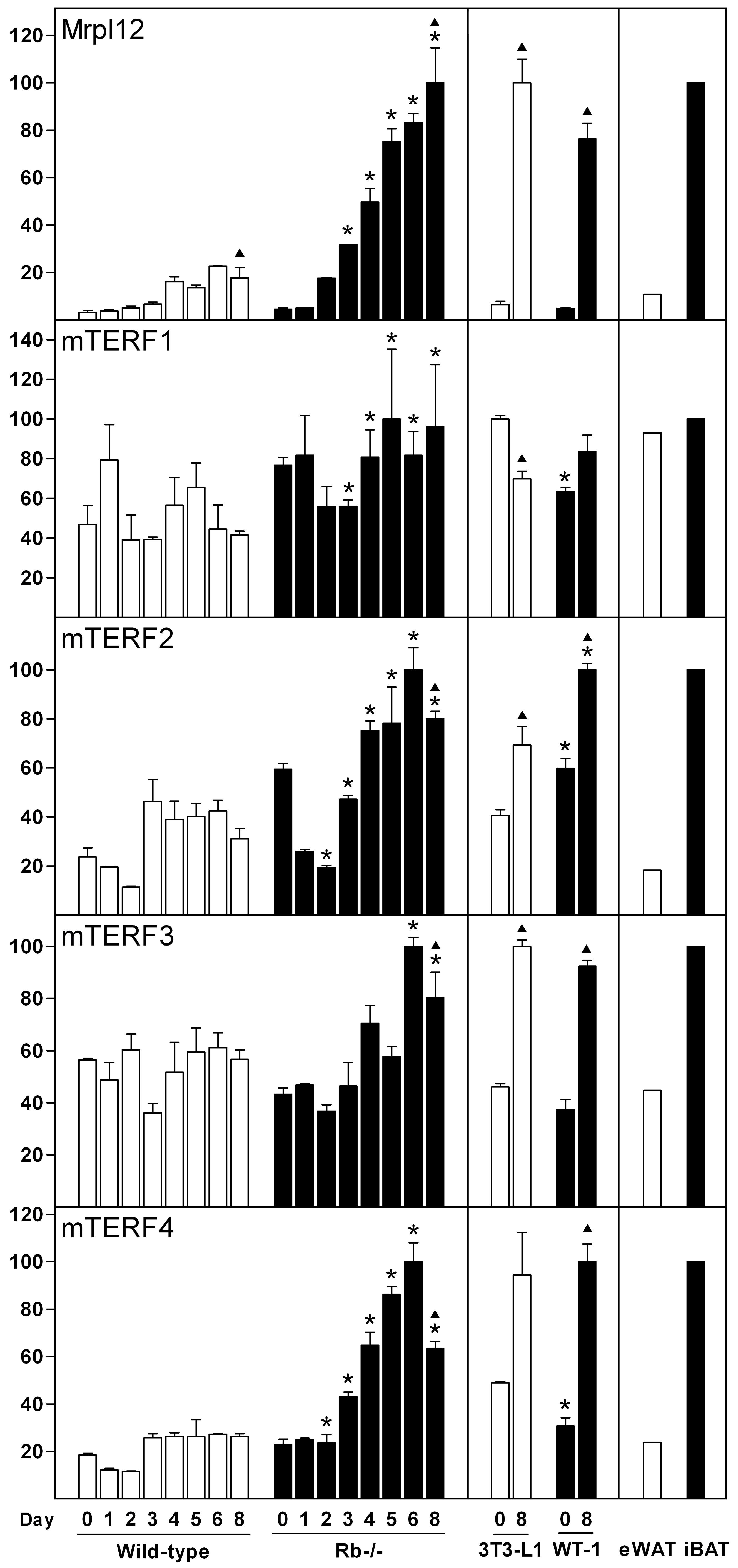

Supplement: Figure S3 — Expression of genes involved in mitochondrial transcription during differentiation of white and brown adipocytes. Cell lines were induced to differentiate as described in “Materials and Methods” and total RNA was harvested at the indicated days of differentiation. In addition, RNA from eWAT and iBAT was included. Expression levels were determined by RT-qPCR and relative expression levels of genes indicated in the figure determined by normalisation to the levels of TBP. In each of the three boxes for the individual genes, the mean of the normalized expression level of the sample with the highest value was set to 100. Error bars represent SEM. Genes measured were Mrpl12 and mTERF1-4. Results from one of two independent cell culture experiments are shown. *, p<0.05 [day X in wild-type MEFs (or 3T3-L1) compared to day X in Rb−/− MEFs (or WT-1)]. Δ, p<0.05 (day 0 vs. day 8 for each of the four cell lines). (0.95 MB TIF) [file pone.0008458.s003.tif]

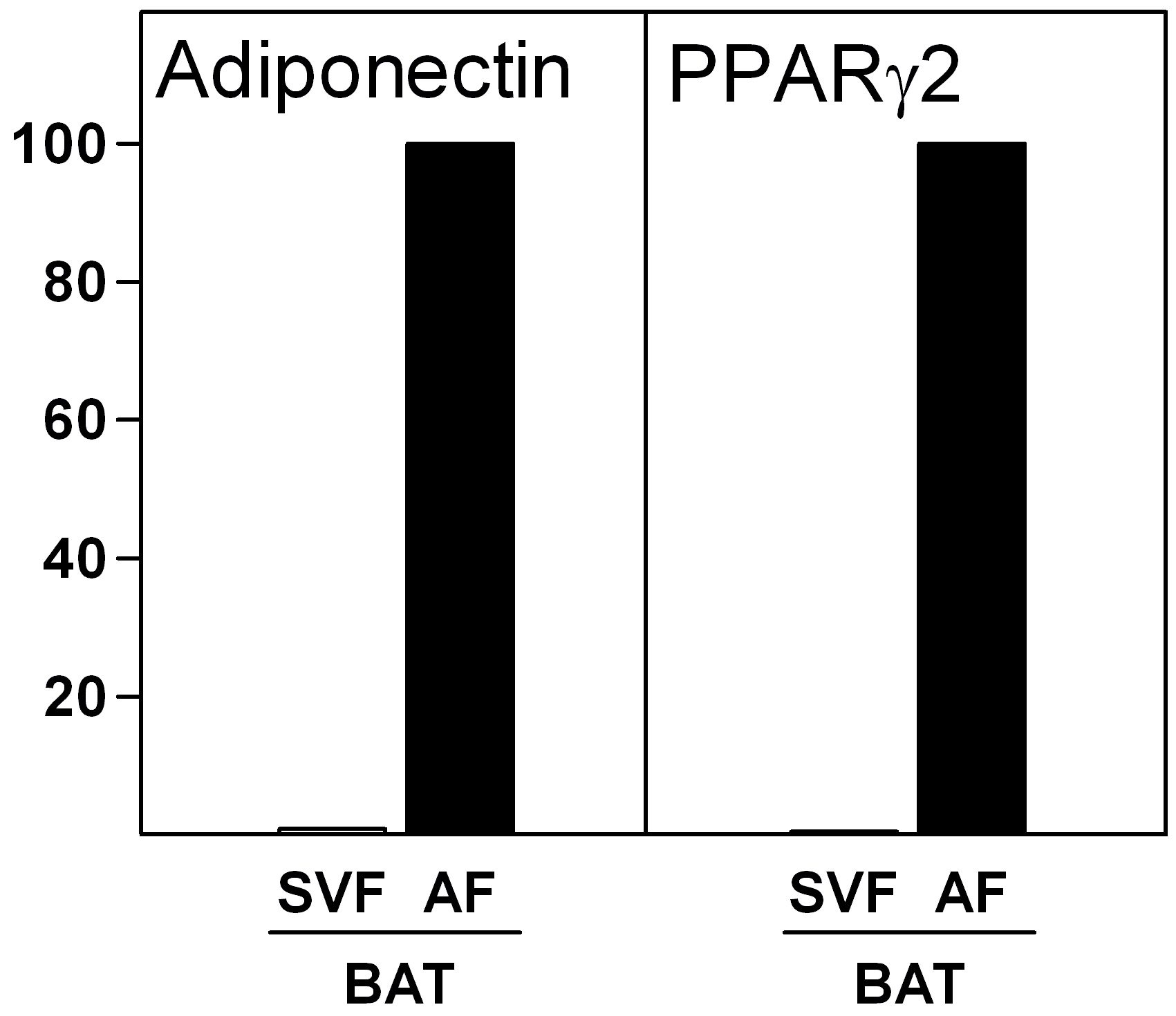

Supplement: Figure S4 — Expression of PPARγ2 and adiponectin in stromal-vascular and adipose fractions of brown adipose tissue. RNA from BAT fractions (pool from 12 mice) was analysed for the expression of PPARγ2 and adiponectin by RT-qPCR. Expression levels were normalised to the expression of 18S rRNA. The highest value of the mean of the normalized expression of PPARγ2 or adiponectin was set to 100. (0.07 MB TIF) [file pone.0008458.s004.tif]

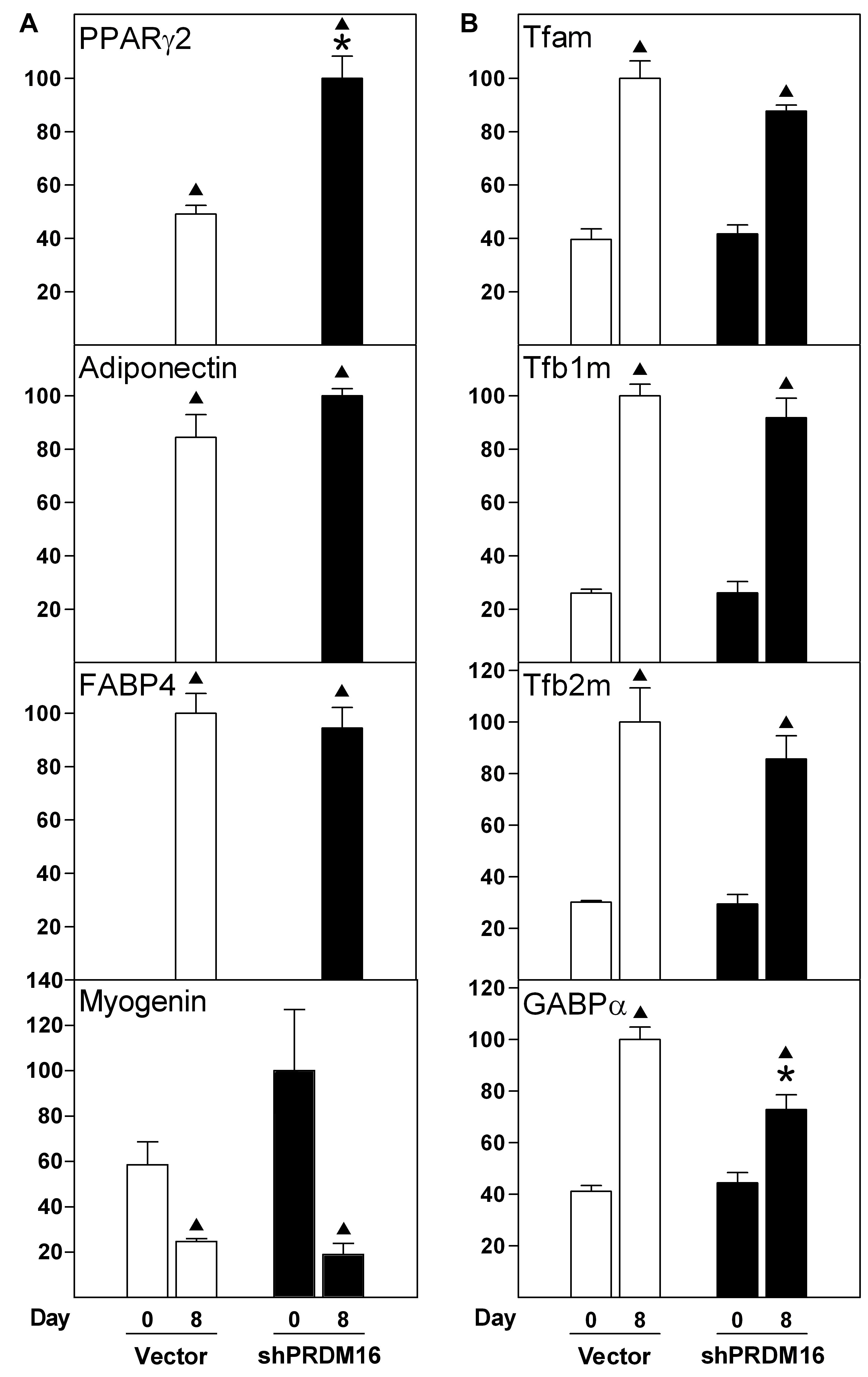

Supplement: Figure S5 — Expression of selected transcription factors after silencing of PRDM16 expression. Rb−/− MEFs were transduced with pSUPER.retro.neo or pSUPER.retro.neo-PRDM16 virus (designated “Vector” and “shPRDM16”, respectively), selected, replated and induced to differentiate as described in “Materials and Methods” and total RNA was harvested at days 0 and 8 of differentiation. Expression levels were determined by RT-qPCR and relative expression levels of genes indicated in the figure determined by normalisation to the levels of TBP. In each of the boxes for the individual genes, the mean of the normalized expression level of the sample with the highest value was set to 100. Error bars represent SEM. (A) Expression of PPARÎ32, adiponectin, FABP4 and myogenin. (B) Expression of Tfam, Tfb1m, Tfb2m and GABPÎ±. Similar results were obtained in three independent experiments. Similar results were obtained in three independent experiments. *, p<0.05 (day X in Vector compared to day X in shPRDM16). Δ, p<0.05 (day 0 vs. day 8 for Vector or shPRDM16). (0.49 MB TIF) [file pone.0008458.s005.tif]
