# Supplementary material for: Dynamic Regulation of Genes Involved in Mitochondrial DNA Replication and Transcription during Mouse Brown Fat Cell Differentiation and Recruitment
Source: PLoS One. 2009 Dec 24;4(12):e8458. doi: 10.1371/journal.pone.0008458 (PMC2809086; doi:10.1371/journal.pone.0008458)
Supplement: Table S1 — Primers used for quantitative PCR (0.09 MB DOC) [file pone.0008458.s006.doc]

**Table S1. Primers used for quantitative PCR**

| **Gene** | **Accession number** | **Reference** | **Product size (bp)** | **Forward primer** | **Reverse primer** |
| --- | --- | --- | --- | --- | --- |
| **Adiponectin** | AY754346 | This study | 102 | AACTTGTGCAGGTTGGATGGC | TTCTCTCCCTTCTCTCCAGGA |
| **C/EBPα** | NM_007678 | Mol. Cell. Biol. 25: 1272-82. 2005. | 127 | TGGACAAGAACAGCAACGAG | TCACTGGTCAACTCCAGCAC |
| **Cidea** | NM_007702 | Diabetes 54: 1726-34. 2005. | 129 | AAAGGGACAGAAATGGACAC | TTGAGACAGCCGAGGAAG |
| **COX II (RNA/mtDNA)** | AF378830 | This study | 100 | AATTGCTCTCCCCTCTCTACG | GTAGCTTCAGTATCATTGGTGC |
| **CPT-1b** | NR_004843 | Cytogenet Genome Res. 105: 215-21. 2004. | 148 | TTTGGGAACCACATCCGCCAA | TTATGCCTGTGAGCTGGCCAC |
| **CS** | NM_026444 | This study | 110 | CTCTTGGGAGCCAAGAACTC | GCCTGCTCCTTAGGTATCAG |
| **Cyc1** | NM_025567 | This study | 91 | CTACCCATGGTCTCATCGTG | GGAAGAGCACACCTGCTTGT |
| **ERRα** | NM_007953 | J. Biol. Chem. 278: 9013-8. 2003. | 130 | GGAGGACGGCAGAAGTACAAA | GCGACACCAGAGCGTTCAC |
| **FABP4** | NM_024406 | Mol. Cell. Biol. 25: 1272-82. 2005. | 111 | TGGAAGCTTGTCTCCAGTGA | AATCCCCATTTACGCTGATG |
| **GABPα** | NM_008065 | This study | 80 | GGCCATAGACATCAATGAACC | CATGAGCATCCAAAGAACACTG |
| **GABPβ** | BC013558 | This study | 131 | GATGTCCCTGGTAGATTTGG | GATGAAGTGGAGAAGTTCCCA |
| **Mrpl12** | NM_027204 | This study | 145 | CGAACTCCTGAAGAAAACATTG | GTGTGTCCGCTCTTTCTGTTT |
| **Myf-5** | NM_008656 | This study | 122 | CAGCAGCTTTGACAGCATCTA | ATGGCTCTGTAGACGTGATCC |
| **MyoD** | NM_010866 | This study | 127 | CCTACAGCTACAAACCCAAGC | GCTCCTCCTTCCTTAGCAGTT |
| **Myogenin** | NM_031189 | This study | 130 | AGTGAATGCAACTCCCACAG | ACGTAAGGGAGTGCAGATTGT |
| **mTERF1** | NM_001013023 | This study | 142 | ATGCGACAGTAAGGACAAGG | CTCCTGCTCATTCGTAACAG |
| **mTERF2** | NM_028832 | This study | 147 | CTGACTGAAGCCCTGGAGA | TGCTGTGAGCTCTGGCTAA |
| **mTERF3** | NM_025547 | This study | 133 | TGCCAGCACAAATAGGACA | AGACAATGGAGGCAGGTCA |
| **mTERF4** | NM_178051 | This study | 111 | GGCACAGCACATCACAGAC | TGTGTGAGTCCCATCCTGA |
| **NRF-1** | NM_010938 | This study | 162 | CAGCACCTTTGGAGAATGTG | CCTGGGTCATTTTGTCCACA |
| **PGC-1α** | NM_008904 | Cell 119:121-35. 2004. | 168 | AGCCGTGACCACTGACAACGAG | GCTGCATGGTTCTGAGTGCTAAG |
| **PGC-1β** | NM_133249 | Cell 119:121-35. 2004. | 142 | CGCTCCAGGAGACTGAATCCAG | CTTGACTACTGTCTGTGAGGC |
| **Polg-A** | BC042571 | This study | 167 | CGCTTCTGCATCAGCATCCA | ACTGCACTGAAAAAGGCGAC |
| **Polg-B** | NM_015810 | This study | 162 | AACAGCAATCAGACACCCAG | GAAGTTAGAGGGACTCATAGC |
| **PolRMT** | BC110697 | Mol. Reprod. Dev. 71: 405-13. 2005. | 263 | GTCTACAGGAGATGTTCACC | CAGGGAGTGGATGAAGTTG |
| **PPARα** | NM_011144 | Endocrinology 146: 3266-76. 2005. | 51 | GTACCACTACGGAGTTCACGCAT | CGCCGAAAGAAGCCCTTAC |
| **PPARγ2** | NM_011146 | J. Biol. Chem. 274: 2386-93. 1999. | 241 | CCAGAGCATGGTGCCTTCGCT | CAGCAACCATTGGGTCAG |
| **PRC** | NM_001081214 | This study | 252 | GTCTAAGGAAGCTGTCCTTCC | CCAGTTCTGGGGCTTGTAACC |
| **PRDM16** | NM_027504 | This study | 93 | TAAGCCTTCACCGTTCTTCAT | CGCAGGTACTTCTCTTTCAGG |
| **RIP140**  **(RNA/mtDNA)** | NM_173440 | This study | 221 | CGGCCTCGAAGGCGTGG | AAACGCACGTCAGTATCGTC |
| **RNase MRP** | NM_026398 | Mol. Reprod. Dev. 71: 405-13. 2005. | 185 | ATACCTCAATGCCTACACTG | TGTACTGGATCAGGAACTTC |
| **RNase MRP RNA** | NR_001460 | This study | 62 | TCGCTCTGAAGGCCTGTTTC | GACTTTCCCCTAGGCGAAAG |
| **SHP** | NM_011850 | NURSA | 102 | CGATCCTCTTCAACCCAGATG | AGGGCTCCAAGACTTCACACA |
| **18S rRNA** | NR_003278 | Cell Metab. 1:231-44. 2005. | 63 | ACCGCAGCTAGGAATAATGGA | GCCTCAGTTCCGAAAACCA |
| **Ssb** | BC028648 | Mol. Reprod. Dev. 71: 405-13. 2005. | 207 | GACATGAGTCTGAAGTAGCC | ACGTCGTCTTCTGACTAACG |
| **TBP** | NM_013684 | J. Biol. Chem. 274: 2386-93. 1999. | 190 | ACCCTTCACCAATGACTCCTATG | ATGATGACTGCAGCAAATCGC |
| **Tfam** | NM_009360 | This study | 175 | CAAGTCAGCTGATGGGTATGG | TTTCCCTGAGCCGAATCATCC |
| **Tfb1m** | BC032930 | This study | 189 | AATTTCCTCCTGGACTTGAGG | AGAGAGCATCTGTAACCCTGG |
| **Tfb2m** | NM_008249 | This study | 201 | GTTCGAATGACTCCTCGTAGG | CATTCTAGCAGCTGTGTCTCC |
| **Twinkle** | AY059385 | This study | 142 | GACGACGACAAGGAACTGC | AGCGATTCTTGGACACCTG |
| **UCP1** | NM_009463 | Cell 119:121-35. 2004. | 151 | GGCATTCAGAGGCAAATCAGCT | CAATGAACACTGCCACACCTC |
