# Supplementary material for: Dynamic Regulation of Genes Involved in Mitochondrial DNA Replication and Transcription during Mouse Brown Fat Cell Differentiation and Recruitment
Source: PLoS One. 2009 Dec 24;4(12):e8458. doi: 10.1371/journal.pone.0008458 (PMC2809086; doi:10.1371/journal.pone.0008458)
Supplement: Table S2 — Normalised expression levels of analysed genes. To allow comparison of expression levels across cell lines and tissues, relevant samples normalised to TBP (gene of interest/TBP) are listed. Expression levels for BAT fractions are not provided, as normalisation in this case was to 18S rRNA. (0.38 MB DOC) [file pone.0008458.s007.doc]

| **Gene** |  | **Wild-type** | **Rb-/-** | **3T3-L1** | **WT-1** | **eWAT** | **iBAT** |  | **iBAT** | | **iWAT** | |
| --- | --- | --- | --- | --- | --- | --- | --- | --- | --- | --- | --- | --- |
|  | **Fig. 1, 4, 5, S1, S2, S3** | | | | | | | **Fig. 6** | | | | |
|  | Day |  |  |  |  |  |  | Day | 28°C | 6°C | 28°C | 6°C |
| **UCP1** | 0  8 | 9.525*10-4  0.0035 | 0.0010  35.7586 | 0.0976  0.2036 | 0.1270  71.105 | 0.0118 | 364.5569 | 1  3 | 117.1718  106.4955 | 2082.1848  3780.0508 | 5.4499  2.3883 | 394.1250  421.5247 |
| **Cidea** | 0  8 | 2.471*10-4  0.0077 | 2.096*10-4  45.4882 | No C_t_  3.6841 | 3.412*10-4  124.9574 | 0.79004 | 955.4258 | 1  3 |  |  |  |  |
| **CPT-1b** | 0  8 | 4.026*10-4  7.726*10-4 | 4.679*10-4  0.03651 | No C_t_  No C_t_ | No C_t_  0.0249 | 1.600*10-4 | 0.0998 | 1  3 | 0.06114  0.11398 | 0.1134  0.1420 | 2.301*10-4  1.910*10-4 | 0.0075  0.0167 |
| **CS** | 0  8 | 0.1979  1.4820 | 0.2549  6.9893 | 0.3946  3.1459 | 0.3916  9.9662 | 1.4743 | 7.2854 | 1  3 | 6.5134  7.3366 | 10.0206  17.5499 | 0.8732  1.3836 | 4.6542  6.4746 |
| **Cyc1** | 0  8 | 1.4265  7.2733 | 0.8760  32.7857 | 5.8624  67.1019 | 9.7189  124.9097 | 4.8232 | 45.5696 |  |  |  |  |  |
| **COX II** | 0  8 | 98.9048  504.8037 | 103.9671  2148.0586 | 2524.8704  5736.6552 | 1192.9123  9726.4207 | 337.7940 | 4299.6395 |  |  |  |  |  |
| **Polg-A** | 0  8 | 0.1020  0.1656 | 0.1563  0.6128 | 0.1548  0.3008 | 0.1618  0.4948 | 0.9559 | 2.0420 |  |  |  |  |  |
| **Polg-B** | 0  8 | 0.1658  0.2647 | 0.2654  0.8272 | 0.4117  0.5618 | 0.3183  0.3997 | 1.0317 | 1.3660 |  |  |  |  |  |
| **Ssb** | 0  8 | 5.2482  5.0923 | 3.4660  8.6340 | 4.6293  5.1085 | 3.2477  5.0437 | 6.9403 | 14.1235 |  |  |  |  |  |
| **Twinkle** | 0  8 | 7.2056  6.3065 | 5.1679  11.9622 | 4.7212  7.8419 | 3.8706  8.0572 | 6.5432 | 21.4068 |  |  |  |  |  |
| **RNase MRP** | 0  8 | 0.2262  1.3176 | 0.3183  4.2546 | 0.1529  0.8728 | 0.1822  1.2090 | 2.1361 | 13.5011 |  |  |  |  |  |
| **RNase MRP RNA** | 0  8 | 9623.6669  6592.9003 | 5313.3589  8838.1347 | 8827.4675  4865.8973 | 3525.5819  3417.0830 | 1807.7757 | 1917.4856 |  |  |  |  |  |
| **PolRMT** | 0  8 | 0.0282  0.0892 | 0.04162  0.28546 | 0.1562  0.5822 | 0.2106  0.5461 | 0.1207 | 0.4061 |  |  |  |  |  |
| **Tfam** | 0  8 | 0.3675  0.4051 | 0.3212  1.1872 | 0.3807  0.7169 | 0.5065  1.5259 | 0.5359 | 1.7654 | 1  3 | 0.6528  0.7429 | 1.2091  1.7932 | 0.1646  0.1286 | 0.9324  0.9272 |
| **Tfb1m** | 0  8 | 0.0594  0.1797 | 0.0881  0.2012 | 0.1557  0.6619 | 0.1786  0.5447 | 0.2398 | 1.1096 | 1  3 | 0.3191  0.2686 | 0.4435  0.5282 | 0.3304  0.5550 | 0.3095  0.3386 |
| **Tfb2m** | 0  8 | 0.0177  0.0320 | 0.0210  0.1021 | 0.0546  0.1336 | 0.0225  0.0936 | 0.1406 | 0.5946 | 1  3 | 0.1037  0.1374 | 0.1891  0.3665 | 9.243*10-4  7.659*10-4 | 0.0945  0.1044 |
| **PGC-1α** | 0  8 | 0.2040  0.4664 | 0.0664  4.7373 | 5.1318  7.2800 | 1.1149  31.760 | 3.7581 | 73.0089 | 1  3 | 7.1692  5.0117 | 114.5704  106.4222 | 2.2460  3.9237 | 33.3910  26.5364 |
| **PGC-1β** | 0  8 | 0.0233  1.0868 | 0.0150  6.0118 | 0.1729  8.6255 | 0.1724  10.779 | 4.8568 | 92.4115 |  |  |  |  |  |
| **PRC** | 0  8 | 0.0016  8.531*10-4 | 0.0016  0.0010 | 0.0027  9.085*10-4 | 0.0014  3.085*10-4 | 4.221*10-4 | 5.687*10-4 |  |  |  |  |  |
| **RIP140** | 0  8 | 0.1754  1.6585 | 0.5185  0.4158 | 0.1956  1.6150 | 0.1872  0.4620 | 1.7411 | 0.2003 |  |  |  |  |  |
| **NRF-1** | 0  8 | 0.4824  0.5631 | 0.5076  0.8386 | 1.5771  1.3257 | 1.8232  2.3329 | 2.3620 | 3.8906 |  |  |  |  |  |
| **GABPα** | 0  8 | 0.2266  0.3048 | 0.2070  0.4881 | 2.6831  2.5829 | 2.5895  4.7706 | 0.3802 | 0.6854 |  |  |  |  |  |
| **GABPβ** | 0  8 | 0.0039  0.0029 | 0.0070  0.0022 | 0.0072  0.0047 | 0.0096  0.0050 | 0.0057 | 0.0105 |  |  |  |  |  |
| **PRDM16** | 0  8 | 3.860*10-4  6.708*10-4 | 0.0061  0.0115 | No C_t_  No C_t_ | 1.495*10-4  9.809*10-4 | 0.0011 | 0.0177 | 1  3 | 0.3042  0.2571 | 0.7941  1.4042 | 0.1109  0.1208 | 0.6154  0.4883 |
| **PPARα** | 0  8 | 0.0075  0.1721 | 6.940*10-4  3.7385 | 0.01671  0.20899 | 0.0393  9.8758 | 1.6586 | 105.4197 |  |  |  |  |  |
| **ERRα** | 0  8 | 0.2149  1.8512 | 0.8716  5.7559 | 7.0799  44.7404 | 4.3316  48.480 | 17.1484 | 133.4356 |  |  |  |  |  |
| **PPARγ2** | 0  8 | 4.47*10-4  0.7891 | 2.47*10-4  0.5406 | 0.0032  1.3556 | 3.097*10-4  0.2536 | 2.4794 | 4.3169 |  |  |  |  |  |
| **C/EBPα** | 0  8 | 0.0146  3.9260 | 0.3326  2.8536 | 0.3988  31.8427 | 8.0398  37.9931 | 129.7868 | 76.1093 |  |  |  |  |  |
| **FABP4** | 0  8 | 0.0347  860.9929 | 0.1434  1163.71 | 9.3658  3470.0070 | 1.8818  3706.1909 | 6793.7857 | 6038.6067 |  |  |  |  |  |
| **Adiponectin** | 0  8 | 8.645*10-4  233.9423 | 0.0409  209.4050 | 0.0075  392,1117 | 0.0432  98.1501 | 982.2865 | 354.5880 |  |  |  |  |  |
| **Myogenin** | 0 | No C_t_ | 7.959*10-4 | 0.0036 | 4.6260 |  |  |  |  |  |  |  |
| **Mrpl12** | 0  8 | 0.0027  0.0148 | 0.0038  0.0837 | 0.0012  0.0185 | 8.882*10-4  0.0141 | 0.0067 | 0.0616 |  |  |  |  |  |
| **mTERF1** | 0  8 | 0.2946  0.2609 | 0.4811  0.6036 | 1.8409  1.2872 | 1.1688  1.5387 | 0.8675 | 0.9330 |  |  |  |  |  |
| **mTERF2** | 0  8 | 0.0325  0.0426 | 0.0817  0.1100 | 0.0849  0.1450 | 0.1247  0.2089 | 0.0497 | 0.2717 |  |  |  |  |  |
| **mTERF3** | 0  8 | 1.3426  1.3499 | 1.0297  1.9127 | 0.5039  1.0950 | 0.4090  1.0122 | 2.0705 | 4.6268 |  |  |  |  |  |
| **mTERF4** | 0  8 | 0.1108  0.1573 | 0.1379  0.3794 | 0.0854  0.1649 | 0.0538  0.1745 | 0.2736 | 1.1487 |  |  |  |  |  |

**Table S2. Normalised expression levels of analysed genes**
